# LipidMem-BigSMILES (LiMBS): A Standardized Representation Language for Lipid Membrane Systems

**DOI:** 10.1021/acs.jctc.6c01274

**Published:** 2026-10-28

**Authors:** Pratima Khadka, Shikha Nangia

**Affiliations:** Department of Biomedical and Chemical Engineering, 2029 Syracuse University, Syracuse, New York 13244, United States

## Abstract

Molecular dynamics simulations of lipid membranes lack a standardized, machine-readable representation, creating significant barriers to reproducibility, data sharing, and AI-driven analyses. Current membrane descriptions are fragmented across software-specific input files, making it difficult to reconstruct published systems or compare results among studies. Here, we introduce LipidMem-BigSMILES (LiMBS), a line notation not a simulation tool or membrane-building program, for lipid membrane simulations that extend SMILES, BigSMILES, and CG-BigSMILES to membrane systems. LiMBS version 1.0 uses a dual-block architecture that separates a system-level descriptor block from a chemical structure block, enabling the independent specification of membrane geometry, composition, environmental conditions, and molecular identity. The framework supports planar bilayers, vesicles, and bacterial outer membranes in atomistic, coarse-grained, and supercoarse-grained resolutions within a unified syntax. We provide a reference tool that reads, checks, and standardizes LiMBS strings, and we show that its output can be handed off to existing membrane-building software for system construction. LiMBS1.0 is human-readable, machine-readable, tool-independent, and compatible with FAIR data principles, providing a standardized description layer for membrane simulations that supports reproducible reporting, interoperable databases, and AI-enabled membrane biophysics.

## Graphical abstract

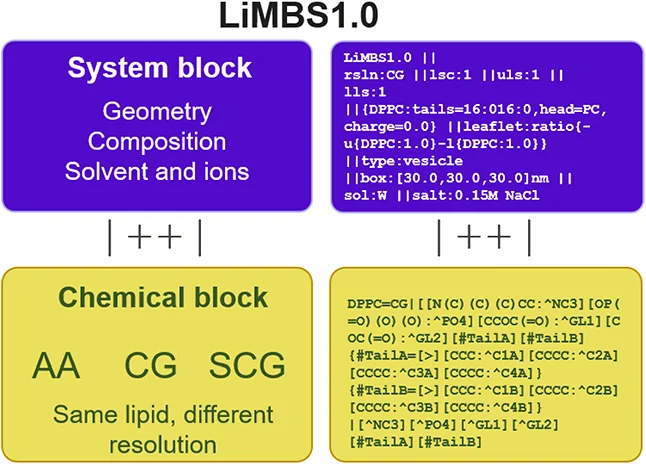

## 1 Introduction

Lipid membranes orchestrate many of life’s essential processes, from nutrient transport and cellular signaling to immune recognition and compartmentalization.^1^ Computational models capture this complexity across scales from angstroms to micrometers, enabling insights unattainable from experiment alone.^2,3^ Yet as the computational membrane biology community has matured—with contributions spanning multiple simulation engines, force fields, membrane builders, and atomistic (AA) and coarse-grained (CG) representations—a critical challenge has emerged: the absence of a standardized, software-independent representation for lipid membrane systems.^4,5^

Lipid membrane systems span diverse geometries and compositions.^1,2^ Planar bilayers, the simplest and most widely studied membrane geometry, remain the workhorses of membrane biophysics.^2,4^ Yet even a seemingly simple planar bilayer can present significant specification ambiguities. Consider a ternary membrane composed of POPC (1-palmitoyl-2-oleoylphosphatidylcholine), POPE (1-palmitoyl-2-oleoyl-*sn*-glycero-3-phosphoethanolamine), and cholesterol at a molar ratio of 40:40:20. Does this ratio apply independently to each leaflet?^6^ How is cholesterol distributed between leaflets? Spherical vesicles introduce additional complexity.^7,8^ A large unilamellar vesicle requires specification of outer and inner leaflet composition, size distribution, and curvature-dependent effects.^8^ More advanced systems—protein-embedded membranes,^9^ asymmetric bacterial outer membranes,^10^ and lipopolysaccharide (LPS) structures in Gram-negative bacteria—demand still more descriptors. Critically, different membrane geometries rely on distinct representation formats: planar bilayers use CHARMM topology files or GROMACS parameter files, whereas vesicles use specialized tools such as TS2CG 2.0, which have incompatible input formats.^11^ No unified notation exists that handles planar bilayers, vesicles, and bacterial membranes with equal clarity.

Despite their ubiquity, membrane models remain nonstandardized.^12^ Researchers wishing to reproduce a published membrane system must decode implicit assumptions scattered across topology files, supplementary methods, and supplementary code. This fragmentation stems from a fundamental gap: while small-molecule chemistry has standardized notations (SMILES,^13,14^ InChI^15^), lipid membranes across simulation packages remain encoded in software-specific formats that hinder data sharing and reproducibility. This challenge is becoming increasingly urgent as machine learning and artificial intelligence (AI) methods increasingly rely on simulation-generated data sets. Without rigorous standardization and FAIR (findable, accessible, interoperable, and reusable) data practices, membrane simulations risk producing inconsistent data sets that undermine future AI-driven discovery.^5,16^

Recent advances in polymer informatics provide a compelling proof-of-concept for how standardized molecular languages can transform an entire field. Leão and colleagues introduced CG-BigSMILES, an extension of BigSMILES that enables the representation of CG polymer structures within a unified notation framework.^17–20^ In this approach, chemical structure, connectivity, and hierarchical organization are encoded within a compact string that is simultaneously human-readable and machine-readable. The resulting standardization facilitates data sharing, database development, interoperability between software platforms, and machine learning applications on polymer properties. More broadly, BigSMILES demonstrated that a well-designed notation can serve not only as a communication tool for researchers but also as a foundation for data-driven discovery. Inspired by this success, we asked whether the same philosophy could be extended to lipid membrane systems. Can a single notation simultaneously describe membrane composition, geometry, topology, and molecular resolution while remaining both human-readable and machine-interpretable? Addressing this question forms the basis for the development of LipidMem-BigSMILES.

LipidMem-BigSMILES (LiMBS) is a standardized, machine-readable notation for lipid membrane systems that extends BigSMILES to capture membrane-specific information. The framework combines two components: a system-level descriptor that specifies membrane geometry, composition, and assembly rules, and a chemical structure block that encodes lipid identity and, when applicable, CG and super-coarse-grained (SCG) mappings. This dual-block design unifies the representation. The same syntax handles both AA representations using SMILES^13^ and CG representations using mapped beads, enabling a direct comparison across resolutions.

## 2 Why Membrane Simulations Need a Standardized Representation

The rapid expansion of membrane simulation studies has exposed a critical gap in standardization and reproducibility.^12^ While computational resources and force field development continue to advance, the methodological practices for constructing, parameterizing, and reporting membrane systems remain highly fragmented.^21^ The reproducibility problems do not stem from the molecular dynamics algorithms themselves but rather from how membrane systems are initially constructed and parameterized,^5,22^ which can produce substantially different physical behaviors.^10,23^ Since membranes exhibit slow collective dynamics, these setup-dependent differences may persist for hundreds of nanoseconds or even microseconds.^12,24^

### 2.1 Membrane Composition

Biological membranes are extraordinarily heterogeneous and contain complex mixtures of phospholipids, sphingolipids, glycolipids, and sterols.^1^ The exact ratio of saturated to unsaturated lipids, cholesterol content, headgroup charge distribution, and acyl chain length can dramatically alter membrane thickness, fluidity, curvature, permeability, and protein interactions.^10,23,24^ Even among studies attempting to model the *same* membrane, substantial differences often exist in lipid composition choices.^12^

### 2.2 Membrane Size

Small bilayer systems are computationally attractive because they reduce simulation costs, but they also introduce substantial finite-size artifacts.^12,24,25^ Membrane undulations, lipid diffusion, and lateral organization depend strongly on the system dimensions. Small membranes suppress long-range fluctuations and artificially constrain lipid dynamics.^26,27^ In systems involving lipid domains or phase separation, an insufficient membrane size can prevent the emergence of biologically relevant behavior.^2^ Unfortunately, there is currently no universally accepted standard for determining adequate membrane dimensions for different biological questions.^10,12,26^

### 2.3 Membrane Symmetry

Lipid symmetry and asymmetry further complicate reproducibility because biological membranes are highly asymmetric, with different lipid species enriched in the inner and outer leaflets.^10,28^ Some studies attempt to incorporate asymmetry, but use different lipid distributions, equilibration strategies, or leaflet-balancing methods.^12,29^ Because leaflet asymmetry strongly affects curvature stress, electrostatics, and membrane mechanics, differences in symmetry assumptions can alter simulation outcomes.^23,30^ When asymmetric membranes are employed, the literature provides inconsistent guidance on how to specify and maintain asymmetry, leading to divergent implementation choices across research groups.^10^

### 2.4 Membrane Geometry

The choice between planar bilayers and vesicular membrane geometries is another important source of variability.^2,12^ Most membrane simulations employ planar bilayers because they are easier to construct and computationally more efficient.^10^ However, many biological membranes are curved rather than flat. Vesicles, endosomes, exosomes, and cellular organelles exhibit curvature-dependent lipid organization and protein interactions that cannot always be captured using planar membranes.^11,31^ When vesicle geometry is employed, researchers must specify additional parameters, including vesicle size, polydispersity, leaflet composition, and assembly method.^32^ These specifications are frequently missing from published reports, making vesicle simulations particularly difficult to reproduce.^10^

### 2.5 Membrane Lipid Resolution

Atomistic simulations provide detailed molecular interactions but are limited in size (typically a few nanometers in each dimension) and duration (typically tens to hundreds of nanoseconds, although microsecond-scale simulations are increasingly accessible).^24,33^ CG models, such as MARTINI,^34–40^ SPICA,^41^ and SIRAH^42^ enable larger and longer simulations by reducing molecular detail. CG membranes may exhibit altered diffusion dynamics or nonphysical aggregation tendencies.^43^ Furthermore, mapping between CG and AA representations is not always unique, introducing additional uncertainty during the backmapping procedures.^44,45^ As a result, studies conducted by using AA and CG models may yield qualitatively different conclusions.^46^ The field lacks standardized methods for specifying exactly which atoms are grouped into which CG beads, making it difficult to validate CG results against AA benchmarks.^36,44^ While MARTINI remains the most widely used CG framework for membrane simulations, alternative lipid force fields, such as SPICA and SIRAH,^41,42^ employ fundamentally different bead-naming schemes and lipid topology definitions.

### 2.6 Simulation Packages

There are multiple molecular dynamics (MD) engines—CHARMM,^47,48^ GROMACS,^49^ AMBER,^50^ NAMD,^51^ OpenMM,^52^ LAMMPS,^53^ and Desmond^54^—that maintain their own format for specifying molecular systems. CHARMM topology files (.top) and GROMACS parameter files (.itp) encode force field parameters and bonded/nonbonded interactions but are verbose and provide no information about membrane lipid composition. AMBER’s prmtop files use semibinary formats that require specialized tools to parse, while NAMD’s PSF/PDB pair must be kept in constant synchronization. LAMMPS embeds assembly logic in input scripts rather than structured data, and emerging platforms like OpenMM rely on complex XML schemas that hinder validation. Beyond engine-specific formats, coordinate files (PDB, GRO, XTC, TRR) capture only atomic positions at single moments with no metadata about how systems were constructed, what assumptions were made, or which force field versions were used.^49,55^ This fragmentation across CHARMM, GROMACS, AMBER, NAMD, and other engines—each with incompatible formats—creates cascading reproducibility challenges that fundamentally prevent standardized data aggregation and cross-platform simulation exchange.^24^ Moreover, chemical drawings in papers provide visual clarity regarding lipid structure but lack specification of the exact mapping schemes for CG models.^13^

No existing representation format simultaneously achieves all necessary criteria for modern membrane biophysics. Table 1 compares existing formats and tools against the requirements for a unified, standardized notation.

**Table 1:**
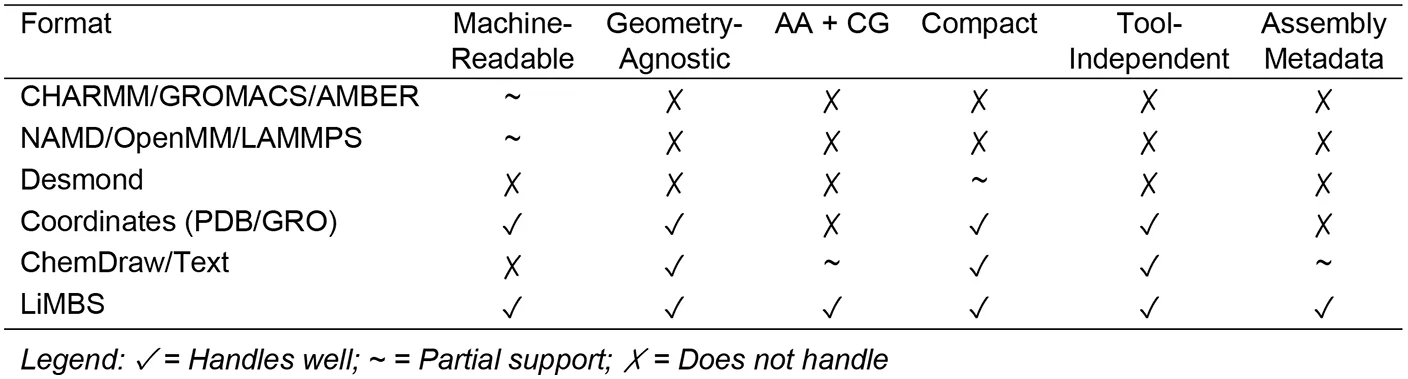
Evaluation of Existing Membrane Representation Methods

These reproducibility issues pose a growing threat to the future application of artificial intelligence (AI) in membrane biophysics and computational biology.^56^ Modern AI and machine learning approaches depend fundamentally on large, high-quality, standardized data sets.^16^ AI models trained on inconsistent data sets may learn artifacts arising from the simulation setup rather than the underlying physical principles.^57^ This lack of standardization threatens the reliability, interpretability, and transferability of AI-driven predictions.^57^

Addressing these challenges will require the broader adoption of FAIR data principles. The membrane simulation community increasingly recognizes the importance of standardized simulation databases, transparent metadata reporting, and open workflows.^16^ Public repositories that store membrane simulation trajectories, input files, force field parameters, and analysis protocols are becoming essential infrastructure for computational biophysics.^58^ Community-wide benchmarking efforts and interoperable databases are critical for future AI applications. Reproducibility, standardization, and FAIR data practices are therefore emerging as central pillars for the next generation of membrane biophysics as the field moves toward AI-assisted molecular discovery.^5,10,16,59^

## 3 LiMBS Design Philosophy

The development of LiMBS was guided by three interrelated design objectives: (i) a notation flexible enough to represent the full diversity of membrane simulation scenarios, (ii) a formal scope that separates membrane specification from simulation execution, and (iii) a reference implementation that validates the notation’s machine-readability in practice.

### 3.1 Design Philosophy

The central innovation of LiMBS, version 1.0, is its dual-block architecture: a system-level descriptor block paired with a chemical structure block. The system block captures membrane-level properties: geometry (planar, vesicular, or other), composition (which lipids, in what ratios, in which leaflets), environmental conditions (solvent, ionic strength), and assembly-relevant metadata (e.g., leaflet counts or ratios) that downstream builders may utilize. The chemical structure captures molecular information: lipid identity, connectivity, and, for CG systems, the explicit mapping rules showing which atoms belong to which beads. This separation reflects a fundamental design insight: two simulations that differ only in membrane geometry or leaflet asymmetry should use identical chemical structure blocks with different system descriptors, whereas two simulations at different molecular resolutions should use the same system descriptor with different chemical blocks.

### 3.2 Resolution Independence

The dual-block architecture enables a direct comparison across modeling resolutions without modifying the underlying membrane specification. Whether a POPC/POPE/cholesterol bilayer is represented in AA or CG resolution, the membrane geometry and composition encoded in the system block remain identical; only the chemical block changes. This resolution independence facilitates systematic, notation-level comparison of the same membrane system across AA and CG specifications, which may support the downstream investigation of resolution-dependent effects. It also provides a natural extension pathway: intermediate resolutions (united-atom, semicoarse-grained) can be incorporated without altering the system block or invalidating existing AA notation. For AA representations, the chemical block encodes all atoms explicitly by using SMILES-style notation. For CG representations, the chemical block uses mapped beads organized hierarchically across layers.

### 3.3 Dual-Layer Mapping for CG Systems

For CG representations, LiMBS employs a hierarchical mapping scheme directly inspired by CG-BigSMILES. Layer 1 defines the mapping between AA substructures and CG beads, capturing the first level of simplification: which atoms are grouped into which bead types (e.g., the choline carbons and nitrogens map to bead NC3). Layer 2 defines the topology of those beads, capturing the bead-level connectivity used in the simulation. This layered design is extensible: a third semantic grouping layer (SCG resolution) can aggregate beads into named functional domains such as HEAD, GLYCEROL, and TAIL, enabling domain-level analytical queries without requiring knowledge of individual bead identifiers. The hierarchical structure preserves the chemical fingerprint of the original molecule—Layer 1 anchors each CG bead to specific atoms, retaining the information that downstream validation, backmapping, and cross-resolution comparison tools would require. By encoding mapping rules explicitly within the notation itself, LiMBS eliminates the ambiguity that currently affects CG mapping schemes described only in chemical drawings or prose.

### 3.4 Relationship to BigSMILES and SMILES

LiMBS builds directly upon the design principles of BigSMILES.^17,18^ It inherits the stochastic object notation for describing repeating structural units and the bonding descriptors for defining molecular connectivity in a compact-string representation. LiMBS extends this framework by adding a system-level descriptor block, a coarse-graining layer syntax, and membrane-specific keywords that capture geometry, leaflet organization, solvation conditions, and ionic composition. The chemical structure portions of LiMBS are fully compatible with BigSMILES and SMILES conventions, ensuring that lipid structures can be exchanged with existing cheminformatics tools and databases without any modification.

### 3.5 Machine Readability

Every element in a LiMBS string has a deterministic syntactic role. System properties are encoded in the outer descriptor block; chemical structures use standard SMILES notation; layer transitions are marked by explicit pipe delimiters; and bead names carry distinct prefixes (^ for CG beads, # for fragments, ^^ for semantic groups). This deterministic syntax enables reliable parsing of membrane geometry, composition, and leaflet distribution without human interpretation, allowing LiMBS strings to serve as standardized upstream inputs for simulation-preparation workflows, database indexing pipelines, and AI-driven search applications.

## 4 Scope of LiMBS1.0

LiMBS1.0 is a line notation, not a membrane construction, parametrization, or simulation method. It captures all descriptors required to unambiguously specify a membrane system for downstream simulation: lipid composition, leaflet asymmetry, membrane geometry (planar or vesicular), molecular resolution (AA, CG, or SCG), solvent model, and ionic conditions. It deliberately excludes force field parameters, simulation protocols (time step, thermostat, barostat), equilibration procedures, and trajectory data. This separation of membrane specification from simulation execution is a foundational design principle: it ensures that a single LiMBS string describes the same membrane system regardless of which simulation engine, force field, or membrane builder is used downstream. The formal grammar governing this syntax is provided in the Supporting Information (S1).

This scope is intentional rather than limiting. Simulation protocols and force field parameters vary widely across engines and research groups and are better captured in companion metadata records or linked data structures—an approach already established by CG-BigSMILES for polymer force fields. LiMBS occupies the complementary role of providing a portable, tool-independent identifier for the membrane system itself, independent of how it is subsequently simulated or analyzed.

### 4.1 Reference Implementation and Validation

To demonstrate that the design principles above yield a deterministic and machine-readable notation in practice, LiMBS is accompanied by a reference software implementation consisting of formal specification files, a parser and validator, a canonicalizer, structured JSON export tools, external identifier linkage, and automated testing utilities (Figure 1).

**Figure 1:**
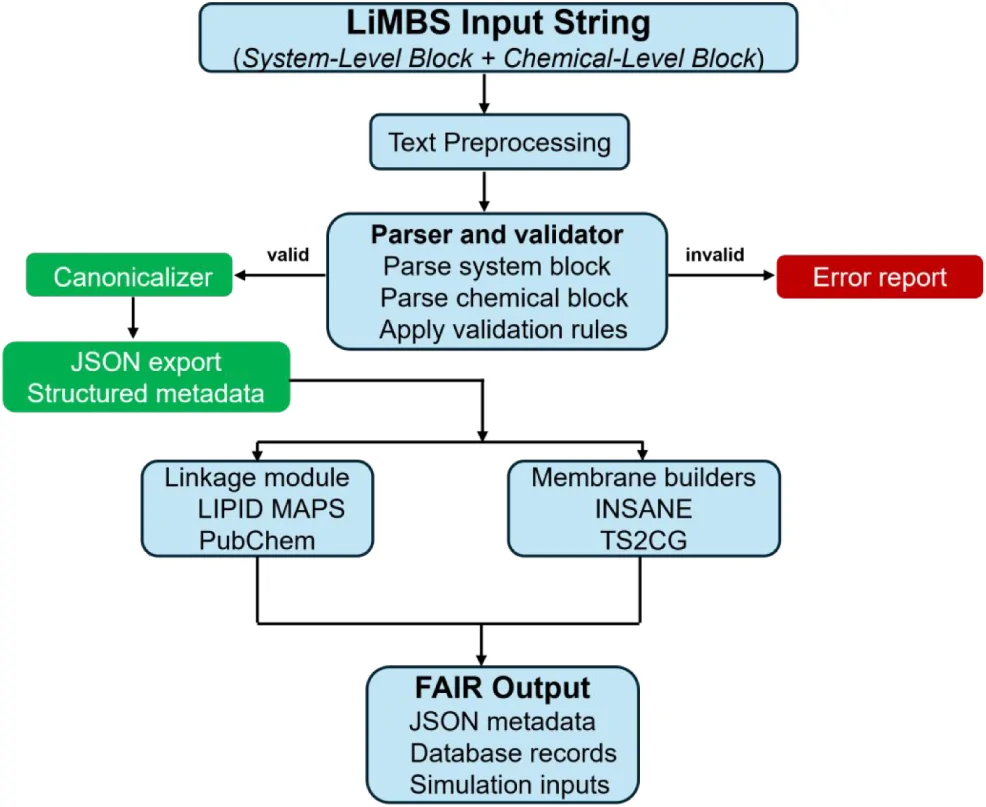
Software architecture of the LiMBS reference implementation. Input LiMBS strings undergo text preprocessing and parser-validator analysis. Valid strings pass to the canonicalizer and JSON exporter; invalid strings are returned with structured error codes. The outputs include canonical LiMBS strings and enriched JSON metadata.

The workflow begins with text preprocessing, where LiMBS strings are normalized to remove formatting inconsistencies prior to deterministic parsing. The parser-validator engine interprets system-level descriptor blocks and species-level chemical blocks while applying both syntax and semantic validation rules (Supporting Information S2). Valid strings pass to the canonicalizer, which generates normalized representations that are suitable for database indexing and deduplication. Malformed inputs are rejected with structured error reporting using a defined error code taxonomy (section S3). Successfully parsed systems are exported as canonical LiMBS strings and enriched JSON metadata, enabling interoperability with external databases and membrane-building tools, including INSANE and TS2CG.

To evaluate the robustness of the reference implementation, we performed quantitative benchmarking across five core functionalities (Table 2). The parser successfully processed all 30 valid membrane systems in the test set (30/30). All 7 deliberately malformed LiMBS strings were correctly identified, and returned specific error codes (7/7). Canonicalization produced identical output for all 10 equivalent string pairs (10/10). Structured JSON export recovered all required metadata fields for 11 representative systems (11/11). For 4 representative systems, LiMBS-derived JSON exports were confirmed to be correctly read by the native input parsers of INSANE and TS2CG (4/4); this confirms format compatibility only and does not constitute validation of the resulting membrane structures. Worked examples of error detection, canonical output, and JSON export are provided in the Supporting Information (S3–S5).

**Table 2:** Quantitative Benchmarking and Validation of the LiMBS Reference Implementation

Benchmark Category	Test Set	Evaluation Metric	Result
Parser validation	30 valid membrane systems	Successful parsing	30/30
Malformed input detection	7 invalid LiMBS strings	Error detection rate	7/7
Canonicalization consistency	10 equiv string pairs	Identical canonical output	10/10
Structured export	11 valid systems	Required metadata recovered	11/11
Export format compatibility	4 test systems	Correctly parsed by INSANE/TS2CG input reader	4/4

## 5 LiMBS Syntax and Grammar

The formal syntax of LiMBS 1.0 is defined here: the delimiter hierarchy separating structural levels, the required field order and the semantics of the system block, the layer conventions for AA and CG chemical blocks, and the validation rules enforced by the parser.

A LiMBS string has two top-level components separated by a three-level delimiter hierarchy (Table 3). The outer |++| delimiter separates the system block from the all-species blocks. Individual species blocks are separated from one another by |+|, while || separates the attribute fields within the blocks. Additionally, in the chemical species block, the single pipe | separates the mapping layers. This three-level hierarchy is strict: delimiters at one level may not appear inside structures governed by a higher level, and each delimiter has exactly one syntactic role. The complete formal grammar in Extended Backus–Naur Form (EBNF) is provided in the Supporting Information (S1).

**Table 3:** LiMBS Delimiter Hierarchy

Delimiter	Role
|++|	Separates system block from species block
|+|	Separates multiple species block
||	Separates fields within the same block
|	Separates mapping layers within a species block

The general structure of a LiMBS string is therefore:

### 5.1 System-Level Descriptor Block


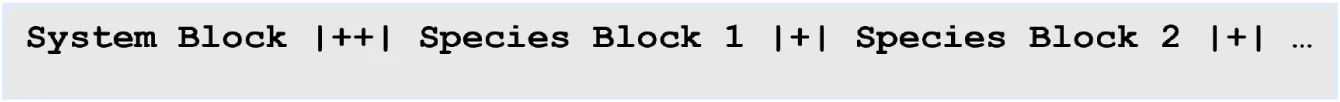


The system block appears first in every LiMBS string and encodes the membrane identity and construction metadata. Fields are separated by ∥ and must appear in a canonical order.

The rsln: field specifies molecular resolution and governs solvent compatibility. AA (atomistic) requires TIP3P, TIP4P, SPC, or SPCE; CG (coarse-grained) requires W; SCG (super coarse-grained) requires WW. A mismatch between rsln: and sol: raises error LMB-E180 or LMB-E181. The three count fields—lsc: (total lipid species), uls: (upper leaflet species), and lls: (lower leaflet species)—enable rapid consistency checking and database filtering by compositional complexity.

The lipid metadata block {Name: tails = ./head = ., charge = .} is a semicolon-delimited list encoding acyl-chain composition (e.g., 16:0–18:1 for palmitoyl-oleoyl), headgroup class (PC, PE, PG, PS, PA, PI, CL, SM, CHOL), and net formal charge for each lipid species. The leaflet block supports two specification modes. For planar bilayers, both count- and ratio-based leaflet specifications are supported. The leaflet:count mode defines absolute integer lipid numbers, whereas leaflet:ratio defines positive relative lipid proportions that must sum to 1.0 independently within each leaflet. For vesicles, ratio-based specifications are used to represent the lipid composition. When ratios are specified, LiMBS records the relative proportions only; determination of the corresponding integer lipid numbers from the membrane dimensions, lipid composition, and area per lipid is performed by the external membrane-building software (e.g., INSANE, TS2CG) at construction time, not by LiMBS1.0. Upper (outer) and lower (inner) leaflet compositions are encoded with -u and -l, respectively, enabling explicit representation of both symmetric and asymmetric membranes.

Supported geometry types (type) are planar and vesicle. Bacterial outer membranes are represented as asymmetric planar bilayers (LPS in the outer leaflet, phospholipids in the inner leaflet) and do not require a separate geometry keyword. Box dimensions (box: [x, y, z] nm) are always in nanometers; the use of any other unit raises LMB-E107. The sol: and salt: M *Formula* fields encode the solvent model and ionic conditions, respectively.

### 5.2 Species-Level Chemical Blocks

Each species block follows the pattern: name = resolution | [layer_1 | layer_2 | layer_3]. The number of layers is governed by resolution: AA uses one layer; CG uses two layers (atom-to-bead mapping and then bead topology); SCG uses three layers (the two CG layers plus a semantic grouping layer). All lipid species referenced in the leaflet block must have corresponding species block definitions; missing definitions raise LMB-E120.

### 5.3 AA Layer

The single layer encodes full molecular connectivity in SMILES-style notation with LiMBS fragment extensions. Fragment notation ({#Name = structure}) allows reusable acyl chain substructures to be defined once and referenced multiple times, reducing redundancy in multicomponent systems. For example, a 16-carbon palmitoyl chain is declared as {#T16 = CCCCCCCCCCCCCCCC} and referenced as [#T16] at each ester linkage position. All fragments must be declared before use; forward references raise LMB-E140.

### 5.4 CG Layer

Layer 1 maps SMILES substructures to named beads using the syntax [SMILES:^BeadName]. Layer 2 contains bead references ([^BeadName]) defining the CG molecular topology. Bead names are case-sensitive and must remain consistent across both layers (LMB-E140). When two beads of the same type occupy distinct structural positions—for example, the two glycerol ester linkages in POPE—positional index suffixes disambiguate them (^GL1 and ^GL2). The D-prefix bead naming convention (^D2A, ^D2B) denotes beads containing a carbon double bond, consistent with Martini nomenclature. Unless otherwise stated, all CG bead assignments and D-prefix bead positions for unsaturated tails in the representative membrane systems follow the MARTINI3 force field itp files. The [>] bonding descriptor at the start of a fragment definition indicates covalent attachment to the preceding group. A complete CG species block for POPE is shown below:
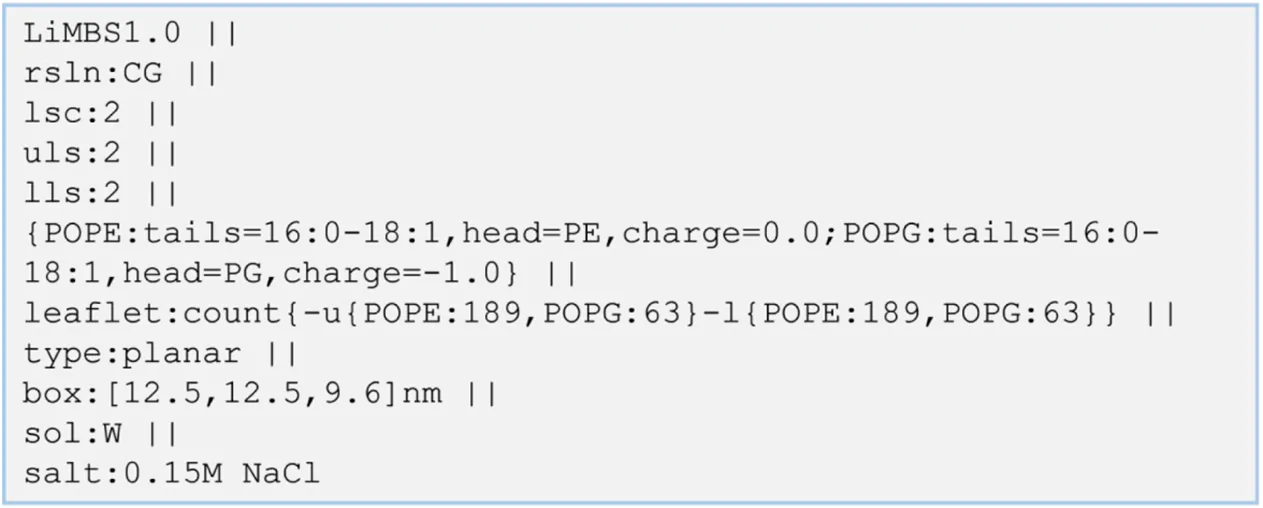


### 5.5 SCG Layer

A third semantic grouping layer aggregates beads into named functional domains using the double caret (^^) prefix. Standard domains for phospholipids are {^^HEAD = [^NC3]­[^PO4]}, {^^GLYCEROL = [^GL1]­[^GL2]}, and {^^TAIL = [#TailA]­[#TailB]}. This layer enables domain-level analytical workflows without requiring knowledge of individual bead identifiers and is valid only when rsln:SCG is declared in the system block.

### 5.6 Validation Rules

The LiMBS parser enforces both syntactic and semantic validation. Key semantic rules are

- **Fragment validation**. All fragments referenced within a species block ([#Name]) must have a corresponding definition ({#Name = ...}) within the same species block. Undefined fragment references raised LMB-E140.
- **Cross-layer bead consistency**. A bead identifier introduced in layer 1 must appear with the same name in layer 2. Bead names may not be created in layer 2 that were not defined in layer 1 (LMB-E140).
- **Leaflet–metadata–species triangle.** Every lipid species named in the leaflet block must appear in the metadata block (LMB-E116) and must have a corresponding species block definition (LMB-E120). Conversely, species blocks defined but not referenced in the leaflet block produce warning LMB-W121.
- **Resolution–solvent compatibility.** The rsln: field and sol: field must be mutually consistent: AA with TIP3P/TIP4P/SPC/SPCE, CG with W, SCG with WW. Mismatches raise LMB-E180 or LMB-E181.
- **Box units**. Box dimensions must be specified in nanometers. Any other unit raises the level of LMB-E107.

A full error code reference and worked examples of valid and invalid LiMBS strings with parser output are provided in the Supporting Information (S3).

## 6 Representative Membrane Systems

To evaluate the expressiveness, scalability, and machine readability of LiMBS, we applied the framework to a representative membrane system spanning increasing structural and compositional complexity. These examples include simple single-component bilayers, curved vesicles, asymmetric biological membranes, and bacterial membrane systems. Together, these examples demonstrate that LiMBS can represent diverse membrane architectures while preserving deterministic syntax and parser compatibility.

**Example 1: Symmetric Planar Membrane**

We first consider an AA-symmetric planar bilayer composed of dipalmitoylphosphatidylcholine (DPPC) in aqueous 0.15 M NaCl (Figure 2). This system serves as a minimal reference example for demonstrating the complete LiMBS syntax.

**Figure 2:**
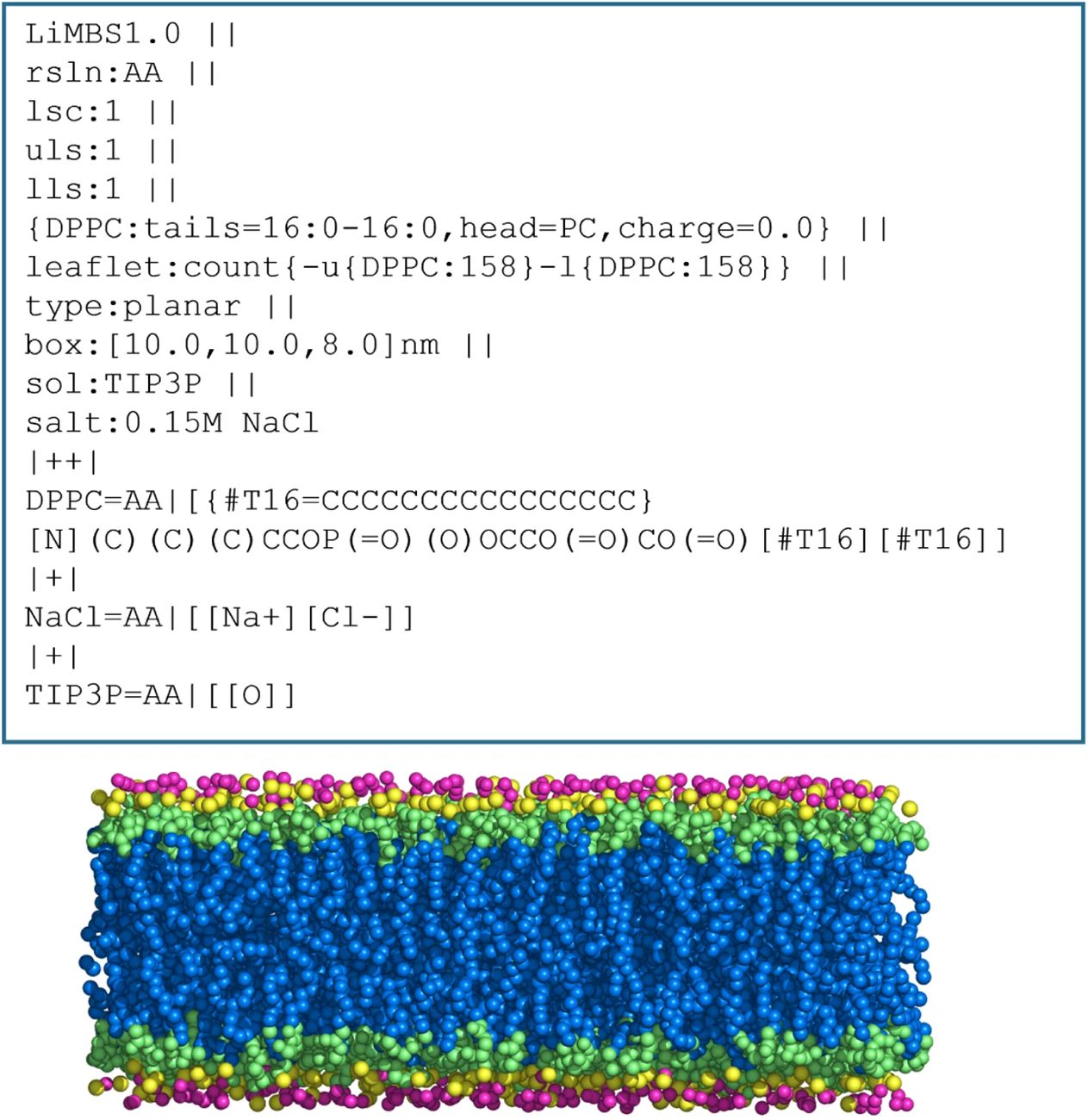
LiMBS string notation for an atomistic DPPC lipid membrane. The membrane rendering shown was generated by supplying the LiMBS-derived JSON export to external membrane-building software for visualization purposes only; it illustrates the specified system and does not constitute simulation validation by LiMBS.

The system block specifies DPPC with a charge state of 0.0 and a phosphatidylcholine (PC) headgroup. The bilayer is symmetric, containing 158 DPPC molecules in each leaflet. The simulation box dimensions are 10 × 10 × 8 nm^3^, corresponding to a membrane with 316 total lipids. Water and 0.15 M NaCl represent the solvent environment. The chemical block defines the full DPPC molecular structure using fragment-based notation to reduce redundancy in repeated acyl chain segments. The LiMBS string notation for this membrane is provided in Supporting Information (S6).

For the CG planar system (Figure 3), the system block remains unchanged, ensuring that membrane composition, geometry, and environmental conditions are preserved across resolutions. The chemical block now contains a CG tag. Layer 1 (before the |) maps AA groups to CG beads: the choline group to NC3, the phosphate group to PO4, the glycerol ester linkages to GL1 and GL2, and the acyl-chain segments to bead types C1A–C4A (#TailA) and C1B–C4B (#TailB). Layer 2 (after the |) provides the simplified bead-level representation while preserving the same molecular topology. This two-layer structure allows users to inspect both the atomistic-to-bead mapping (Layer 1) and the resulting CG topology (Layer 2).

**Figure 3:**
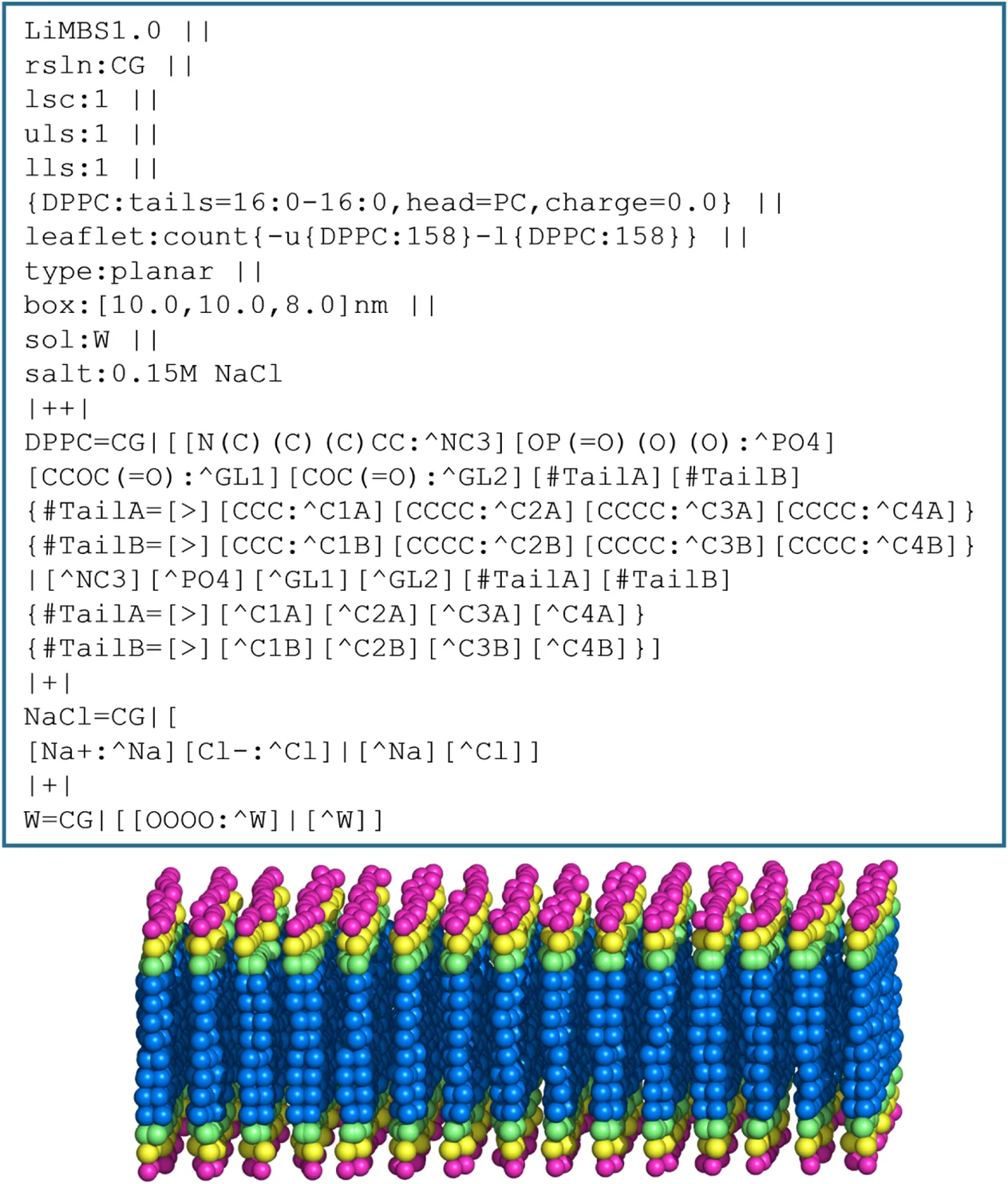
LiMBS string notation for a coarse-grained DPPC lipid membrane. Membrane rendering generated externally, as described in Figure 2.

Comparison of LiMBS notations in Figures 2 and 3 shows that AA and CG descriptions share the same system block, ensuring that only the molecular representation changes while the underlying membrane specification remains identical. The LiMBS string notation for this membrane is provided in the Supporting Information (S7).

Furthermore, the DPPC bilayer can be described at super-CG or SCG resolution using a three-layer structure (Figure 4). The LiMBS string notation for this membrane is provided in the Supporting Information (S8). The system block specifies one lipid species (DPPC) with identical compositions in both leaflets, resulting in a symmetric bilayer with sol:WW. The third semantic grouping layer uses the ^^ prefix to aggregate beads into functional domains: {^|^HEAD = [^NC3]­[^PO4]}, {^|^GLYCEROL = [^GL1]­[^GL2]}, and {^|^TAIL = [#TailA]­[#TailB]}.

**Figure 4:**
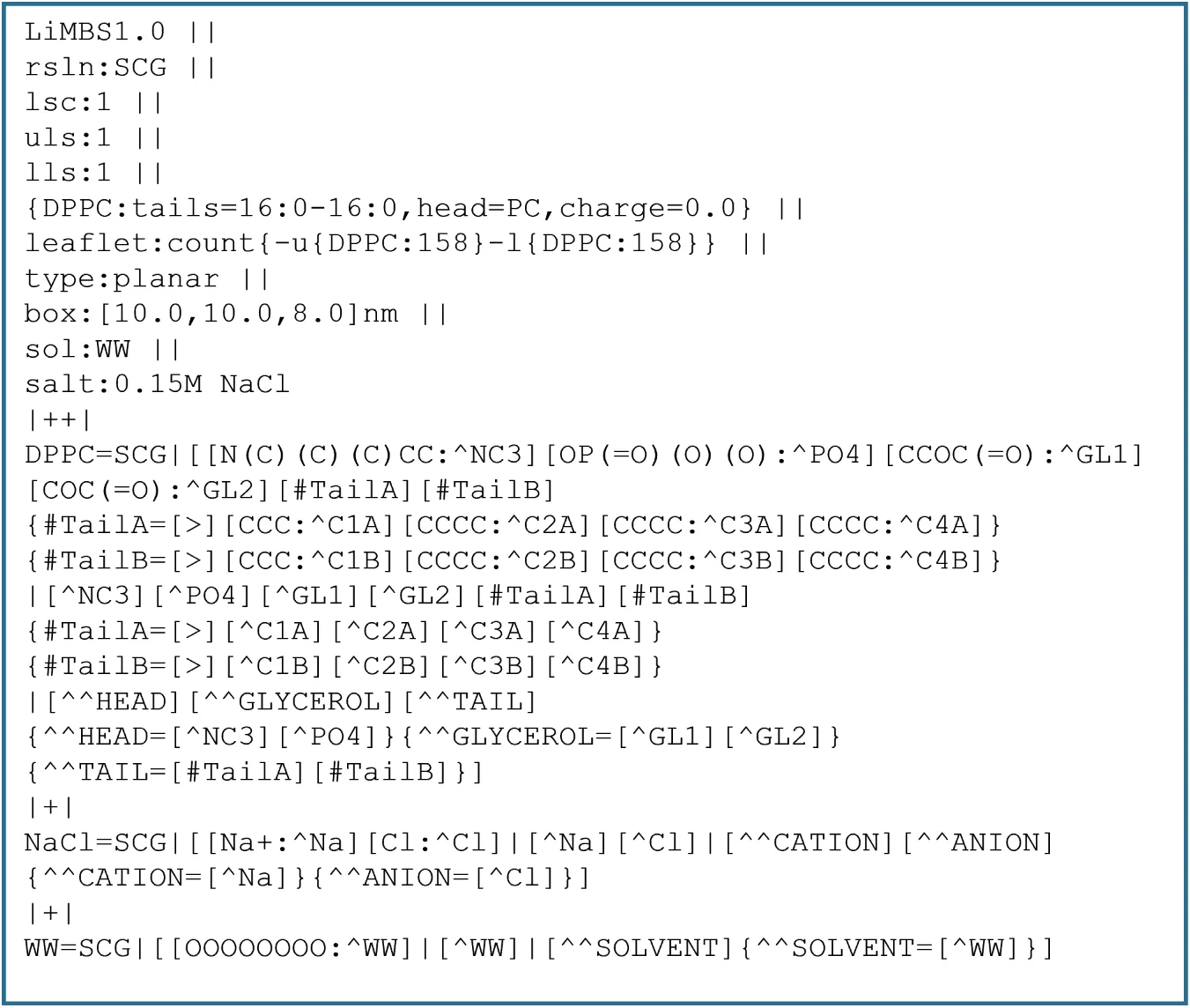
LiMBS string notation for a DPPC SCG lipid membrane.

**Example 2: Simple Lipid Vesicle**

To illustrate LiMBS syntax for curved membrane systems, consider a simple vesicle composed of DPPC in CG resolution. Figure 5 shows the corresponding LiMBS specification together with the resulting vesicular membrane structure. The system block defines DPPC with a neutral charge and a phosphatidylcholine (PC) headgroup. Unlike the bilayer case, where lipid counts are explicitly defined, the leaflet specification uses DPPC:1 for both outer and inner leaflets. In this context, the value represents a relative proportion rather than the lipid count, allowing the total lipid count to be determined implicitly from the vesicle geometry and APL. The type keyword is set to vesicle, indicating a closed, curved membrane structure rather than a planar bilayer. The simulation box is defined as 30 × 30 × 30 nm^3^, providing sufficient volume to accommodate the vesicle and its surrounding environment.

**Figure 5:**
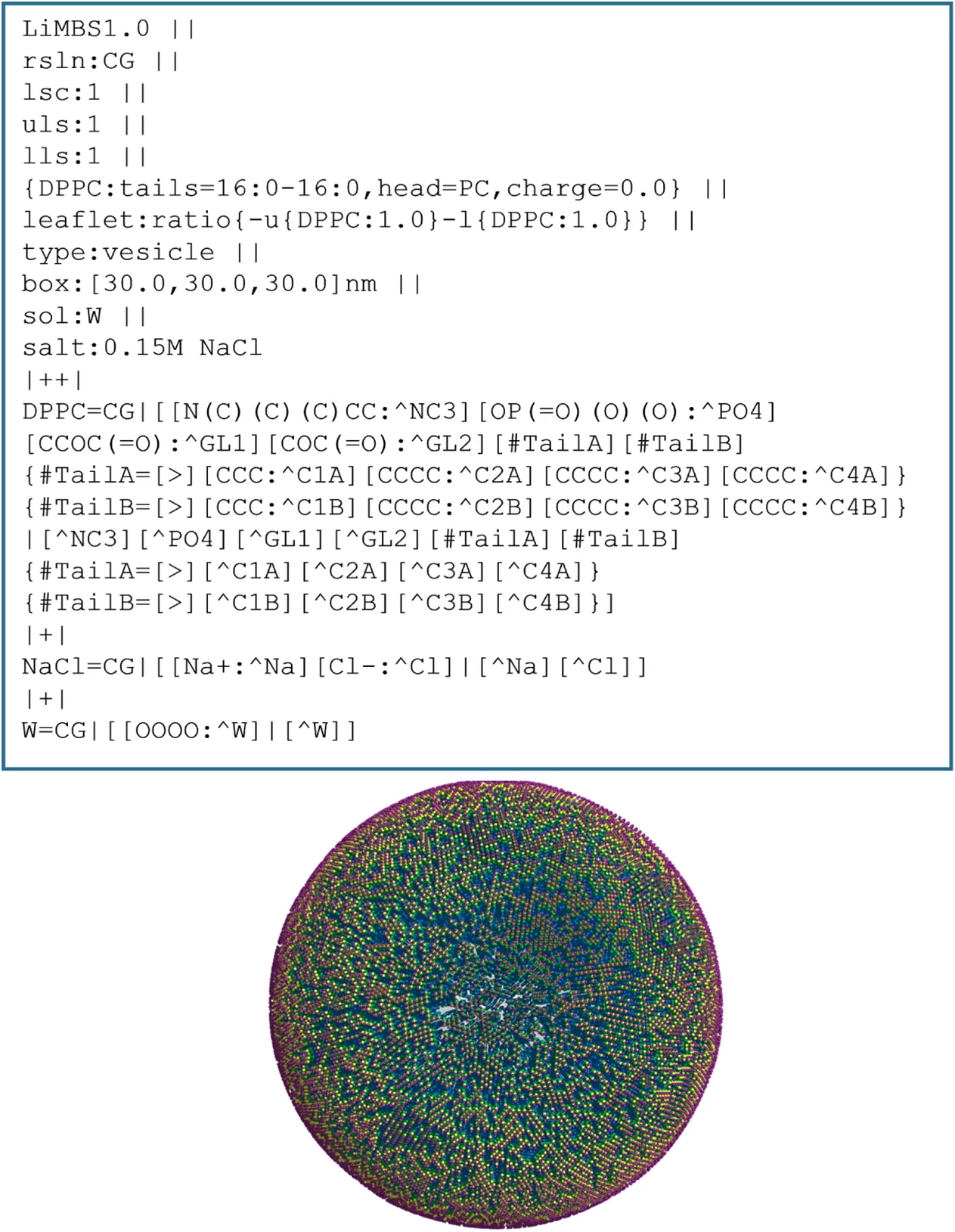
LiMBS string notation for a DPPC vesicle lipid membrane. Membrane rendering generated externally, as described in Figure 2.

The chemical block follows the same two-layer CG representation used in example 1. Layer 1 maps AA fragments to CG beads, and layer 2 presents the simplified bead-level topology, preserving the same connectivity while omitting AA detail. The LiMBS notation of this vesicle in the CG representation is provided in the Supporting Information (S9).

**Example 3: Symmetric Bilayer with Cholesterol**

To demonstrate that LiMBS can encode published AA membrane simulations and enable direct cross-resolution comparison, we represent a previously published DOPC/cholesterol bilayer system.^60^ In that work, the effect of cholesterol on unsaturated DOPC bilayer stiffness was investigated using an atomistic molecular dynamics simulation with the CHARMM36 force field at physiologically relevant cholesterol concentrations.^60^ We encode the 10 mol % cholesterol system containing 145 DOPC and 16 cholesterol molecules per leaflet (322 total lipids) in a 10 × 10 × 8 nm^3^ simulation box with TIP3P water and 0.15 M NaCl, corresponding to the liquid-disordered membrane phase (Figure 6). The LiMBS notation of this membrane is provided in the AA and CG representations in the Supporting Information (S10 and S11).

**Figure 6:**
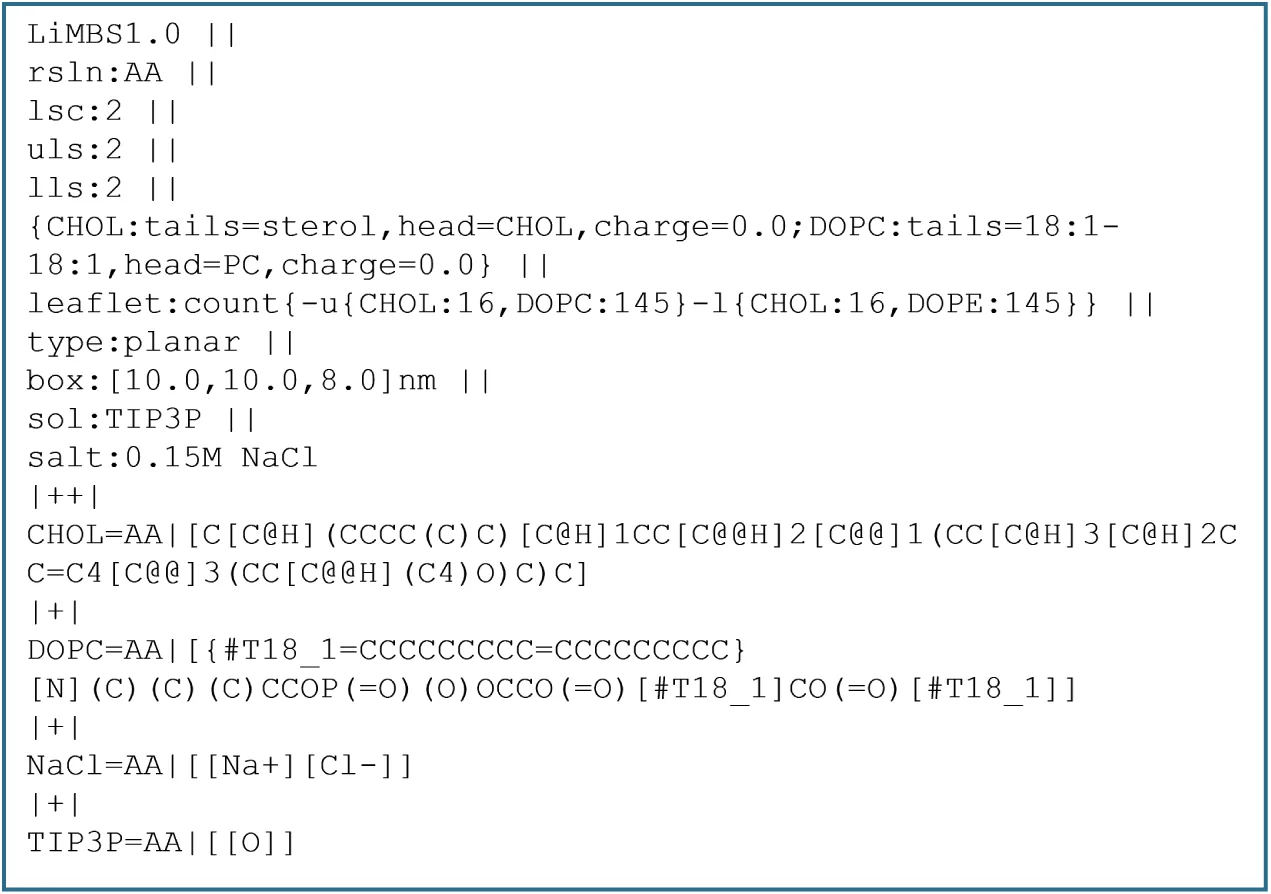
LiMBS string notation for an atomistic DOPC and cholesterol membrane.

**Example 4: Bacterial Outer Membrane**

To demonstrate the LiMBS representation of a biologically relevant asymmetric membrane, we constructed a descriptor for the outer membrane of *E. coli*. Figure 7 shows the corresponding LiMBS system specification and the resulting asymmetric bacterial outer membrane.

**Figure 7:**
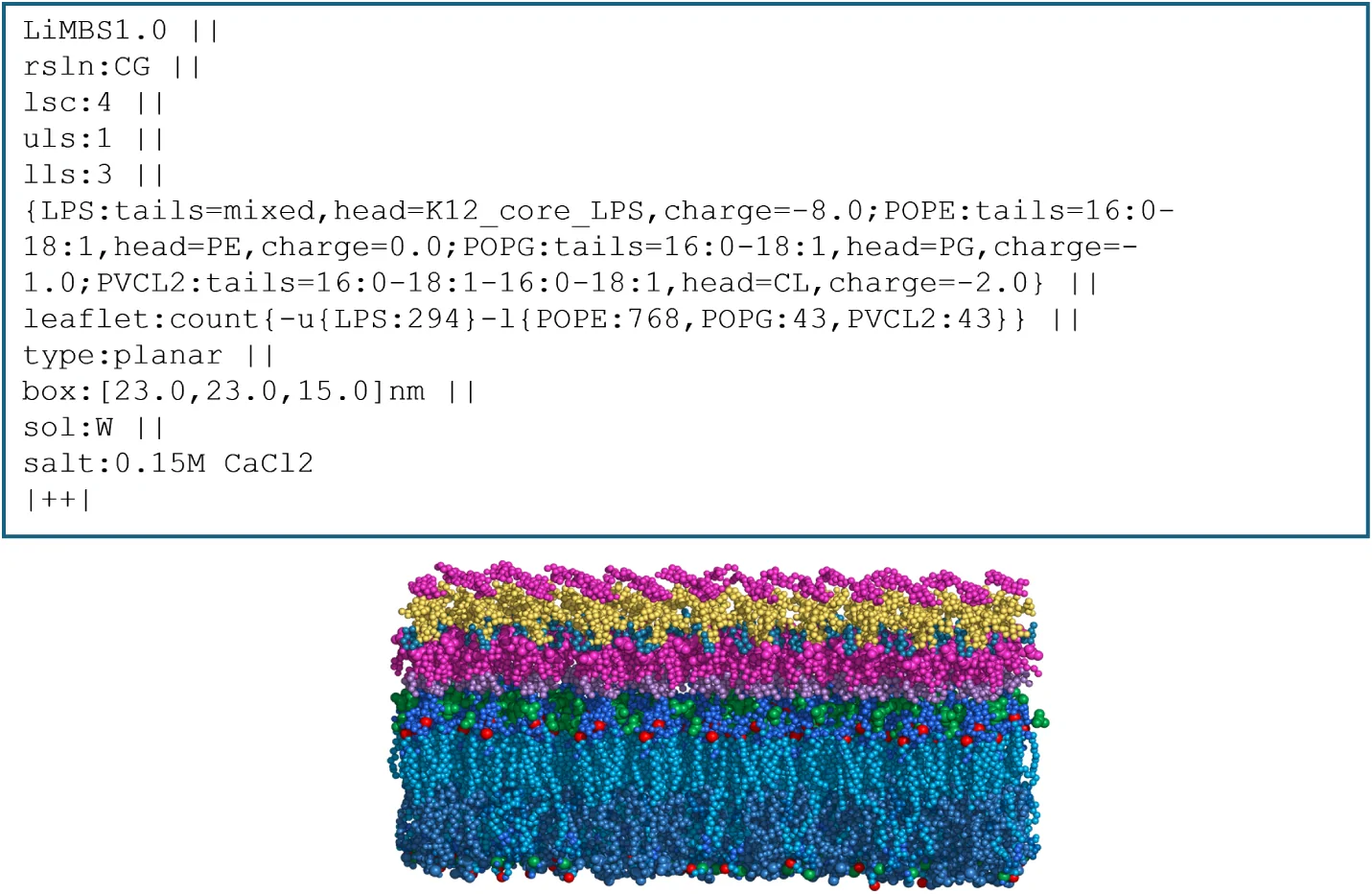
LiMBS string notation for an *E. coli* outer membrane model. Membrane rendering generated externally, as described in Figure 2.

This system captures the characteristic asymmetry of Gram-negative bacterial outer membranes, in which the outer leaflet is composed primarily of lipopolysaccharides (LPSs), whereas the inner leaflet contains phospholipids. Only system-level parameters are presented here; the chemical structure blocks in AA (S12) and CG (S13) representations are provided in the Supporting Information.

**Example 5: Complex Multicomponent Membrane**

To demonstrate the LiMBS representation of a complex multicomponent membrane, we constructed a LiMBS descriptor for a 10-component CG membrane system. Figure 8 shows the corresponding system-level LiMBS specification, while complete species-level chemical block definitions are provided in the Supporting Information (S14).

**Figure 8:**
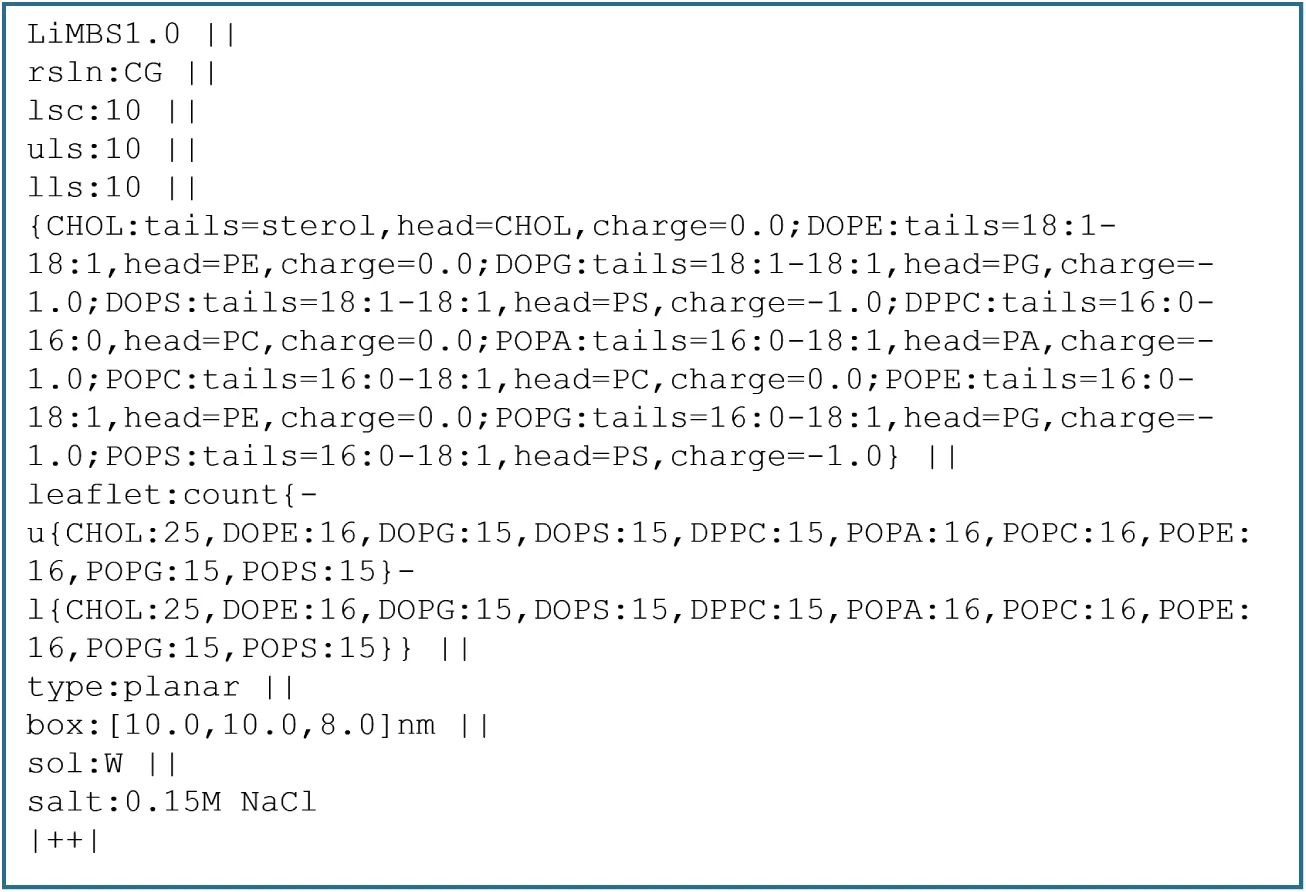
LiMBS string notation for a complex asymmetric membrane.

**Example 6: Representation of the SPICA Membrane System**

Although the examples presented in this work primarily use the MARTINI CG force field, LiMBS1.0 is not restricted to MARTINI-based representations. To demonstrate its broader applicability, we applied LiMBS to the SPICA CG force field,^41^ which employs distinct bead-naming conventions (Supporting Information, S15). Despite the force-field-specific differences, LiMBS1.0 can represent both MARTINI and non-MARTINI CG membrane systems using the same core grammar, highlighting its utility as a force-field-agnostic membrane description language.

In this representation, native SPICA bead identifiers were preserved to maintain consistency with the original force-field topology. Bead labels such as NC, PH, GL, EST1, EST2, GL2, OAD, PEP, and tail-specific identifiers (C11–C26) correspond directly to the SPICA topology definitions. The symbol following ^ denotes the native SPICA bead identifier used for each mapped chemical segment (Figure 9), demonstrating that LiMBS preserves force-field-specific topology information without requiring modification of the underlying syntax.

**Figure 9:**
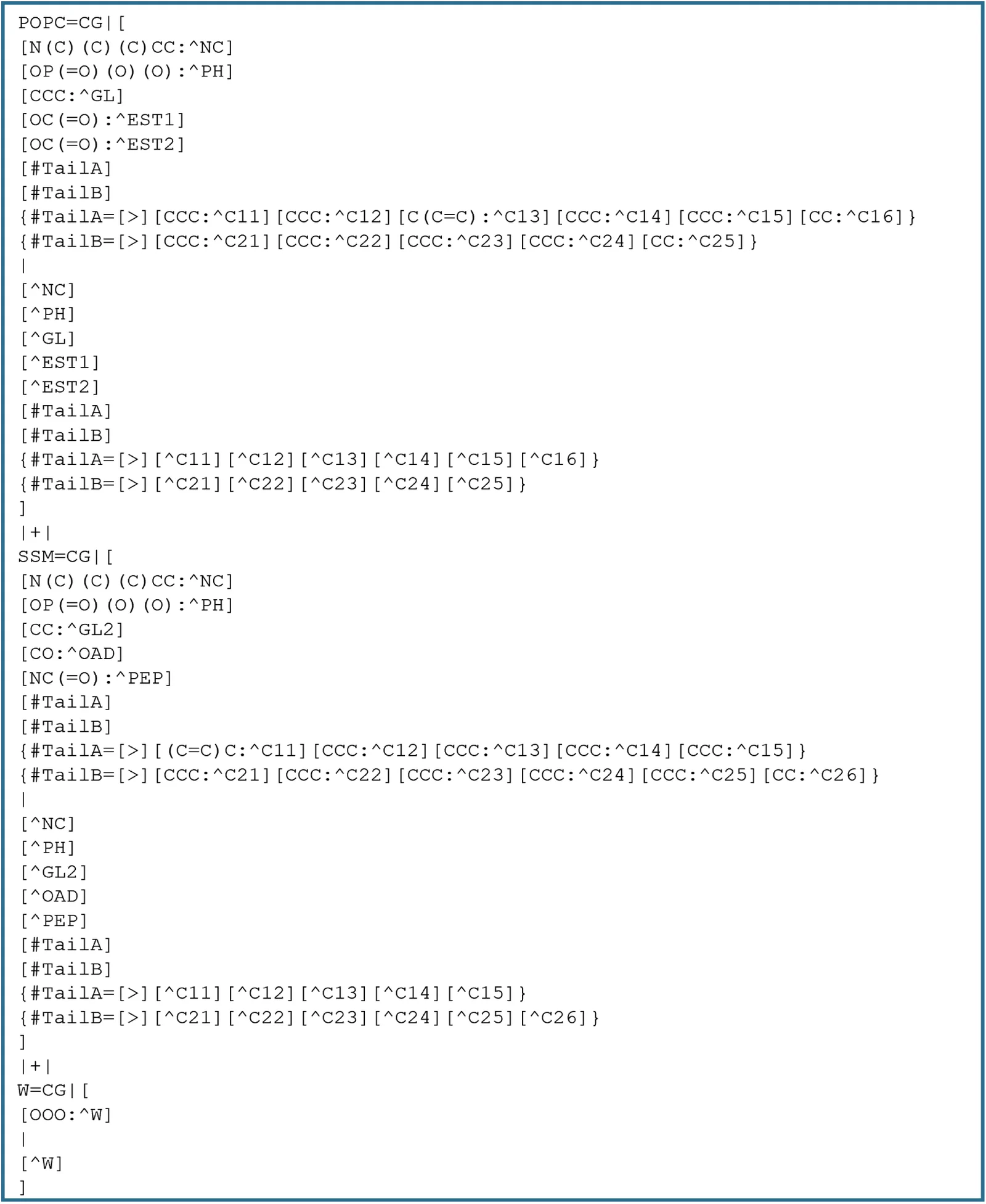
LiMBS representation of a SPICA coarse-grained membrane system containing POPC, SSM, and water.

Together, these examples demonstrate that LiMBS provides a unified framework for representing membrane systems across the full range of structural and compositional complexity encountered in biomolecular simulations, from simple single-component bilayers to asymmetric bacterial outer membranes. LiMBS1.0 is intentionally scoped to the membrane specification and construction metadata; force field parameters, simulation protocols, and trajectory data are deliberately excluded and are better captured in companion data structures, following the precedent established by CG-BigSMILES for polymer systems. Future versions will extend the framework to glycocalyx structures, membrane-associated polymers, lipid nanoparticles, and curvature fields as community needs evolve. As membrane simulations continue to increase in scale and importance for data-driven discovery, standardized representations such as LiMBS provide the common language needed for reproducible research, interoperable databases, and AI-enabled membrane informatics.

## 7 Conclusions

LipidMem-BigSMILES (LiMBS) introduces a standardized, machine-readable representation language for lipid membrane systems. By separating the membrane architecture from molecular representation through a dual-block design, LiMBS1.0 provides a unified framework for describing membrane composition, geometry, leaflet organization, environmental conditions, and molecular identity across a wide range of membrane systems. The notation supports planar bilayers, vesicles, asymmetric membranes, and bacterial outer membranes while remaining compatible with both AA and CG representations. Beyond providing compact notation, LiMBS1.0 addresses a longstanding challenge in computational membrane science: the absence of a common language for describing membrane systems that is independent of simulation software and molecular resolution. The framework enables reproducible membrane specification, facilitates interoperability across simulation workflows, and supports the development of searchable membrane databases and standardized metadata. These capabilities align naturally with the FAIR data principles and provide a foundation for automated workflows and data integration. As membrane simulations continue to increase in scale, complexity, and importance for data-driven discovery, standardized representations will become an essential infrastructure for computational membrane science. By providing a common, software-independent language for membrane systems, LiMBS1.0 establishes a foundation for reproducible simulations, interoperable databases, and future AI-enabled membrane informatics.

## Supplementary Material



## Data Availability

The LiMBS 1.0 script and all examples are available in the GitHub repository at https://github.com/NangiaLab/LiMBS. There is no restriction on the use of the script.

## Notes

Formal LiMBS 1.0 syntax and validation rules; representative AA, CG, and SCG membrane examples; INSANE and TS2CG construction workflows; ratio-based membrane construction; and the reusable lipid registry and CG template library (PDF)
