# Supplementary material for: LipidMem-BigSMILES (LiMBS): A Standardized Representation Language for Lipid Membrane Systems
Source: J Chem Theory Comput. 2026 Oct 28;22(21):11634–46. doi: 10.1021/acs.jctc.6c01274 (PMC13757749; doi:10.1021/acs.jctc.6c01274)
Supplement: Supplementary file 1 [file ct6c01274_si_001.pdf]

Supporting Information

LipidMem-BigSMILES (LiMBS): A Standardized Representation Language for Lipid Membrane Systems

Pratima Khadka and Shikha Nangia*
Department of Biomedical and Chemical Engineering, Syracuse University, Syracuse, NY 13244, USA

*Address for correspondence:
Dr. Shikha Nangia
343 Link Hall
Department of Biomedical and Chemical Engineering
Syracuse University, Syracuse, NY 13244, USA
Phone (315) 443 0571 **|** Email: snangia@syr.edu
ORCID 0000-0003-1170-8461

Table of Contents

S1. Formal Syntax Definition (EBNF)-LiMBS1.0 3
S2. LiMBS parser 5
S3. Invalid Examples 6
S4. Canonicalization Examples 9
S5. Enriched JSON Output Examples 11
S6. DPPC Bilayer (Atomistic) 14
S7. DPPC Bilayer (Coarse-grained) 15
S8. DPPC Bilayer-Super Coarse-Grained (SCG) 16
S9. DPPC Vesicle (Coarse-grained) 17
S10. DOPC-Cholesterol (Atomistic) 18
S11. DOPC-Cholesterol (Coarse-grained) 19
S12. Bacterial Outer Membrane of *E. coli* (Atomistic) 21
S13. Bacterial Outer Membrane of *E. coli* (Coarse-Grained) 23
S14. Complex Multicomponent Membrane 26
S15. Representation of SPICA Membrane System 31
S16. Ratio-Based Planar Membrane 33
S17. Reusable lipid definition and template library 35

S1. Formal Syntax Definition (EBNF)-LiMBS1.0

LiMBS1.0 grammar in Extended Backus-Naur Form (EBNF). The grammar describes the structure accepted by the LiMBS parser after preprocessing and is complemented by semantic validation rules that check consistency among system metadata, leaflet composition, molecular species definitions, solvent, salt, fragment references, and resolution level.

Symbol	Meaning
=	Is defined as
;	End of rule
,	sequence
[…]	Optional expression
{….}	Zero or more repetitions
(…)	grouping
"…"	Literal terminal
?...?	Prose-defined terminal

```
LiMBS_String  ::= System_Block "|++|" Chemical_Block ;
Chemical_Block ::= Species_Block ;

System_Block  ::= 'LiMBS1.0' '||'
                  'rsln:' Resolution '||'
                  'lsc:' PosInt '||'
                  'uls:' PosInt '||'
                  'lls:' PosInt '||'
                  Metadata_Block '||'
                  Leaflet_Block '||'
                  'type:' Geometry '||'
                  Box_Block '||'
                  'sol:' SolventName ['||' Salt_Block ] ;
Resolution     ::= 'AA' | 'CG' | 'SCG' ;
Geometry       ::= 'planar' | 'vesicle' ;
SolventName    ::= 'TIP3P' | 'TIP4P' | 'SPC' | 'SPCE' (* AA *)
                 | 'W'                        (* CG *)
                 | 'WW' ;                     (* SCG *)
Metadata_Block ::= '{' LipidMeta (';' LipidMeta)* '}' ;
LipidMeta      ::= Name ':tails=' TailSpec ',head=' HeadSpec ',charge=' SignedNum ;
TailSpec       ::= 'sterol'
                 | 'mixed'
                 | AcylChain ('-' AcylChain)* ;
AcylChain       ::= PosInt ':' NonNegInt ;
NonNegInt      ::= Digit+ ;
HeadSpec       ::= Name ;
Leaflet_Block  ::= Count_Leaflet | Ratio_Leaflet ;
Count_Leaflet  ::= 'leaflet:count{' '-u{' CountComp '}' '-l{' CountComp '}}' ;
Ratio_Leaflet  ::= 'leaflet:ratio{' '-u{' RatioComp '}' '-l{' RatioComp '}}' ;
CountComp      ::= Name ':' PosInt
                  (',' Name ':' PosInt)* ;
RatioComp     ::= Name ':' Number
```

```
                  (',' Name ':' Number)* ;


Species_Block ::= SpeciesDef ('|+|' SpeciesDef)* ;
SpeciesDef    ::= Name '=' Resolution '|[' LayerContent ']' ;
(* AA:1 layer    CG:2 layers   SCG: 3 layers *)
LayerContent  ::= Layer ('|' Layer)* ;
Layer         ::= (BracketAtom | FragmentRef | FragmentDef
                | SemanticDef  | SemanticRef | WhiteSpace)* ;
BracketAtom   ::= '[' SMILES ':^' BeadName ']' ;
FragmentRef   ::= '[#' Name ']' ;
FragmentDef   ::= '{#' Name '=' '[>]' (BracketAtom | FragmentRef)+ '}' ;
SemanticDef   ::= '{^^' Name '='  (BeadRef  | FragmentRef)+ '}';
BeadRef     ::= '[^' BeadName ']' ;
SemanticRef ::= '[^^' Name ']' ;
BeadName    ::=  Letter (Letter | Digit | '_')*;

Box_Block   ::= 'box:[' Number ',' Number ',' Number ']nm' ;
Salt_Block  ::= 'salt:' Number 'M' WhiteSpace SaltSpecies ;
SaltSpecies ::= ? nonempty salt species or chemical formula ? ;
SMILES      ::= ? molecular substructure text permitted by LiMBS ? ;
WhiteSpace  ::= ? one or more whitespace characters ? ;

Name        ::= Letter (Letter | Digit | '_')* ;
PosInt      ::= NonZeroDigit Digit* ;
Number      ::= Digit+ ['.' Digit+];
SignedNum   ::= ['-' | '+'] Number ;
Letter      ::= [A-Za-z] ;
Digit       ::= [0-9] ;
NonZeroDigit ::= [1-9] ;
```

Leaflet composition semantics. Leaflet: count specifies user-defined positive integer lipid populations, whereas leaflet: ratio specifies positive relative lipid proportions. In leaflet: ratio mode, the values must sum to 1.0 independently within the upper and lower leaflets. A lipid identifier may appear only once within each composition. Builder compatibility is system-specific in LiMBS1.0. For planar CG membranes, the current LiMBS-INSANE interface supports both leaflet: count and leaflet: ratio. For CG vesicles, the current LiMBS-TS2CG interface uses leaflet: ratio.

S2. LiMBS parser

Input String:

```
LiMBS1.0 ||
rsln:AA ||
lsc:1 ||
uls:1 ||
lls:1 ||
{DPPC:tails=16:0-16:0,head=PC,charge=0.0} ||
leaflet:count{-u{DPPC:158}-l{DPPC:158}} ||
type:planar ||
box:[10.0,10.0,8.0]nm ||
sol:TIP3P ||
salt:0.15M NaCl
|++|
DPPC=AA|[
{#T16=CCCCCCCCCCCCCCCC}
[N](C)(C)(C)CCOP(=O)(O)OCCO(=O)[#T16]CO(=O)[#T16]
]
|+|
NaCl=AA|[
[Na+][Cl-]
]
|+|
TIP3P=AA|[
[O]
]
```

Parse Tree

```
LiMBS_String
├── System_Block
│   ├── Version: LiMBS1.0
│   ├── Resolution: AA
│   ├── Lipid species count: 1
│   ├── Upper leaflet species count: 1
│   ├── Lower leaflet species count: 1
│   ├── Metadata_Block
│   ├── Leaflet_Block
│   ├── Geometry: planar
│   ├── Box: [10.0,10.0,8.0] nm
│   ├── Solvent: TIP3P
│   └── Salt: 0.15 M NaCl
└── Chemical_Block
    └── Species_Block
        └── SpeciesDef: DPPC=AA
            ├── FragmentDef: #T16
            └── Layer 1: atomistic structure
```

S3. Invalid Examples

Example 1: Space in box field

```
LiMBS1.0 ||
rsln:CG ||
lsc:1 ||
uls:1 ||
lls:1 ||
{DPPC:tails=16:0-16:0,head=PC,charge=0.0} ||
leaflet:count{-u{DPPC:158}-l{DPPC:158}} ||
type:planar ||
box: [10.0,10.0,8.0]nm ||
sol:W ||
salt:0.15M NaCl
|++|
```

Validate output: Validation failed.

LMB-E040: Invalid box block. Expected: box:[10.0,10.0,8.0]nm

Example 2: Missing species block

```
LiMBS1.0 ||
rsln:CG ||
lsc:2 ||
uls:2 ||
lls:2 ||
{DOPC:tails=18:1-18:1,head=PC,charge=0.0;POPC:tails=16:0-
18:1,head=PC,charge=0.0} ||
leaflet:count{-u{DOPC:100,POPC:58}-l{DOPC:100,POPC:58}} ||
type:planar ||
box:[10.0,10.0,8.0]nm ||
sol:W ||
salt:0.15M NaCl
|++|
```

Validate output:

Validation failed.

Error code reference

<table>
<tr><th>Code</th><th>Meaning</th><th>Common cause</th></tr>
<tr><td>LMB-E000</td><td>Empty input or empty block</td><td>File is blank or |++| missing</td></tr>
<tr><td>LMB-E001</td><td>Does not begin with LiMBS1.0</td><td>First line wrong or file truncated</td></tr>
<tr><td>LMB-E002</td><td>Not exactly one |++| separator</td><td>Duplicate |++|, or missing entirely</td></tr>
<tr><td>LMB-E003</td><td>Duplicate system field</td><td>Two {lipid} blocks, or field appears twice</td></tr>
<tr><td>LMB-E004</td><td>Invalid resolution</td><td>rsln: CG(space after colon) instead of rsln:CG</td></tr>
<tr><td>LMB-E010/11</td><td>Invalid lipid metadata</td><td>Missing commas, wrong format, unrecognized text</td></tr>
<tr><td>LMB-E012</td><td>Duplicate lipid in metadata</td><td>Same name listed twice inside{}</td></tr>
<tr><td>LMB-E020</td><td>Invalid leaflet block</td><td>Wrong syntax</td></tr>
<tr><td>LMB-E024</td><td>Duplicate lipid in the same leaflet composition</td><td>Same lipid identifier is listed more than once within the same -u{} or -l{} block</td></tr>
<tr><td>LMB-E040</td><td>Invalid box block</td><td>Space before bracket, wrong unit, missing values</td></tr>
<tr><td>LMB-E060/61</td><td>Invalid salt field</td><td>Wrong format or missing species name</td></tr>
<tr><td>LMB-E071</td><td>Invalid species definition</td><td>Missing=or resolution keyword</td></tr>
<tr><td>LMB-E072</td><td>Duplicate species</td><td>Same name defined twice after |++|</td></tr>
<tr><td>LMB-E080/81</td><td>Unbalanced brackets</td><td>Missing [or] in species body</td></tr>
<tr><td>LMB-E107</td><td>Box unit not nm</td><td>Angstrom or other unit used</td></tr>
<tr><td>LMB-E108</td><td>Invalid box dimension</td><td>One or more box dimensions are zero or negative</td></tr>
<tr><td>LMB-E116</td><td>Leaflet lipid missing from metadata</td><td>Lipid in leaflet:count not in {} block</td></tr>
<tr><td>LMB-E117</td><td>Invalid leaflet ratio sum</td><td>Leaflet:ratio values in the upper or lower leaflet do not sum to 1.0</td></tr>
<tr><td>LMB-E120</td><td>Leaflet lipid missing species definition</td><td>Lipid in leaflets has no matching species block</td></tr>
<tr><td>LMB-E140</td><td>Undefined fragment used</td><td>#TailA used but {#TailA=..} not defined</td></tr>
<tr><td>LMB-E160</td><td>Undefined semantic group</td><td>^^HEAD used but {^^HEAD=..} not defined</td></tr>
<tr><td>LMB-W121</td><td>Solvent has no species definition</td><td>sol:W declared but W=CG|[…] missing</td></tr>
<tr><td>LMB-W122</td><td>Salt ion not found in species blocks</td><td>NaCl declared but no Na/Cl bead labels found</td></tr>
<tr><td>LMB-W130</td><td>Species resolution mismatch</td><td>DPPC defined as AA but system resolution is CG</td></tr>
<tr><td>LMB-W151</td><td>CG species have only one layer</td><td>Missing | between canonical and mapping layers</td></tr>
<tr><td>LMB-W153</td><td>SCG species has fewer than three layers</td><td>Missing | between layers</td></tr>
<tr><td>LMB-E180</td><td>Invalid solvent for CG resolution</td><td>CG system uses a solvent tother than W</td></tr>
<tr><td>LMB-E181</td><td>Invalid solvent for SCG resolution</td><td>SCG system uses a solvent other than WW</td></tr>
<tr><td>LMB-W180</td><td>Unrecognized AA solvent</td><td>AA system specifies a solvent other than TIP3P, TIP4P, SPC, or SPCE.</td></tr>
</table>

S4. Canonicalization Examples

The –canonical flag produces normalized, unique LiMBS strings. Canonical ordering rules: lipids alphabetical in metadata; leaflet compositions alphabetical; species blocks in order lipids (alphabetical) > salt > solvent; keyword has no spaces (rsln:CG, box:[x,y,z]nm).

Example 1: CG DPPC bilayer canonical output:

```
LiMBS1.0 ||
rsln:CG ||
lsc:1 ||
uls:1 ||
lls:1 ||
{DPPC:tails=16:0-16:0,head=PC,charge=0.0} ||
leaflet:count{-u{DPPC:158}-l{DPPC:158}} ||
type:planar ||
box:[10.0,10.0,8.0]nm ||
sol:W ||
salt:0.15M NaCl
|++|
DPPC=CG|[ [N(C)(C)(C)CC:^NC3] [OP(=O)(O)(O):^PO4] [CCOC(=O):^GL1]
[COC(=O):^GL2] [#TailA] [#TailB]
{#TailA=[>][CCC:^C1A][CCCC:^C2A][CCCC:^C3A][CCCC:^C4A]}
{#TailB=[>][CCC:^C1B][CCCC:^C2B][CCCC:^C3B][CCCC:^C4B]} | [^NC3] [^PO4]
[^GL1] [^GL2] [#TailA] [#TailB] {#TailA=[>][^C1A][^C2A][^C3A][^C4A]}
{#TailB=[>][^C1B][^C2B][^C3B][^C4B]} ]
|+|
NaCl=CG|[ [Na+:^Na][Cl-:^Cl] | [^Na][^Cl] ]
|+|
W=CG|[ [OOOO:^W] | [^W] ]
```

Example 2- CG DOPC/CHOL bilayer canonical output

```
LiMBS1.0 ||
rsln:CG ||
lsc:2 ||
uls:2 ||
lls:2 ||
{CHOL:tails=sterol,head=CHOL,charge=0.0;DOPC:tails=18:1-
18:1,head=PC,charge=0.0} ||
leaflet:count{-u{CHOL:16,DOPC:145}-l{CHOL:16,DOPC:145}} ||
type:planar ||
box:[10.0,10.0,8.0]nm ||
sol:W ||
salt:0.15M NaCl
|++|
CHOL=CG|[ [CCC(O):^ROH] [#TailA] [#TailB]
{#TailA=[>][(C=C)C:^R1][CCC:^R2][CCC:^R3][CC:^R4][CC:^R5][CCC:^R6]}
{#TailB=[>][CCC:^C1][CCCCC:^C2]} | [^ROH] [#TailA] [#TailB]
{#TailA=[>][^R1][^R2][^R3][^R4][^R5][^R6]} {#TailB=[>][^C1][^C2]} ]
|+|
DOPC=CG|[ [N(C)(C)(C)CC:^NC3] [OP(=O)(O)(O):^PO4] [CCOC(=O):^GL1]
[COC(=O):^GL2] [#TailA] [#TailB]
{#TailA=[>][CCCCC:^C1A][CC(C=C):^D2A][CCCC:^C3A][CCCC:^C4A]}
{#TailB=[>][CCCCC:^C1B][CC(C=C):^D2B][CCCC:^C3B][CCCC:^C4B]} | [^NC3] [^PO4]
```

```
[^GL1] [^GL2] [#TailA] [#TailB] {#TailA=[>][^C1A][^D2A][^C3A][^C4A]}
{#TailB=[>][^C1B][^D2B][^C3B][^C4B]} ]
|+|
NaCl=CG|[ [Na+:^Na][Cl-:^Cl] | [^Na][^Cl] ]
|+|
W=CG|[ [OOOO:^W] | [^W] ]
```

S5. Enriched JSON Output Examples

DPPC_CG vesicle -enriched JSON


```
{
  "version": "1.0",
  "resolution": "CG",
  "lipid_species_count": 1,
  "upper_leaflet_species_count": 1,
  "lower_leaflet_species_count": 1,
  "lipids": {
    "DPPC": {
      "tails": "16:0-16:0",
      "head": "PC",
      "charge": 0.0
    }
  },
  "leaflets": {
    "mode": "ratio",
    "upper": {
      "DPPC": 1.0
    },
    "lower": {
      "DPPC": 1.0
    }
  },
  "membrane_type": "vesicle",
  "box": {
    "values": [
      30.0,
      30.0,
      30.0
    ],
    "unit": "nm"
  },
  "solvent": "W",
  "salt": {
    "concentration": 0.15,
    "unit": "M",
    "species_text": "NaCl",
    "species": [
      "NaCl"
    ],
    "formula": {
      "Na": 1,
      "Cl": 1
    },
    "ions": {
      "Na": 1,
      "Cl": 1
    }
  },
  "species_blocks": {
    "DPPC": {
      "name": "DPPC",
      "resolution": "CG",
      "body": "[ [N(C)(C)(C)CC:^NC3] [OP(=O)(O)(O):^PO4] [CCOC(=O):^GL1]
[COC(=O):^GL2] [#TailA] [#TailB]
```

```
{#TailA=[>][CCC:^C1A][CCCC:^C2A][CCCC:^C3A][CCCC:^C4A]}
{#TailB=[>][CCC:^C1B][CCCC:^C2B][CCCC:^C3B][CCCC:^C4B]} | [^NC3] [^PO4]
[^GL1] [^GL2] [#TailA] [#TailB] {#TailA=[>][^C1A][^C2A][^C3A][^C4A]}
{#TailB=[>][^C1B][^C2B][^C3B][^C4B]} ]",
      "layer_count": 2,
      "layers": [
        {
          "index": 1,
          "body": "[N(C)(C)(C)CC:^NC3] [OP(=O)(O)(O):^PO4] [CCOC(=O):^GL1]
[COC(=O):^GL2] [#TailA] [#TailB]
{#TailA=[>][CCC:^C1A][CCCC:^C2A][CCCC:^C3A][CCCC:^C4A]}
{#TailB=[>][CCC:^C1B][CCCC:^C2B][CCCC:^C3B][CCCC:^C4B]}"
        },
        {
          "index": 2,
          "body": "[^NC3] [^PO4] [^GL1] [^GL2] [#TailA] [#TailB]
{#TailA=[>][^C1A][^C2A][^C3A][^C4A]} {#TailB=[>][^C1B][^C2B][^C3B][^C4B]}"
        }
      ],
      "fragments_defined": [
        "TailA",
        "TailB"
      ],
      "fragments_used": [
        "TailA",
        "TailB"
      ],
      "beads": [
        "C1A",
        "C1B",
        "C2A",
        "C2B",
        "C3A",
        "C3B",
        "C4A",
        "C4B",
        "GL1",
        "GL2",
        "NC3",
        "PO4"
      ],
      "semantic_groups": [],
      "semantic_group_definitions": {}
    },
    "NaCl": {
      "name": "NaCl",
      "resolution": "CG",
      "body": "[ [Na+:^Na][Cl-:^Cl] | [^Na][^Cl] ]",
      "layer_count": 2,
      "layers": [
        {
          "index": 1,
          "body": "[Na+:^Na][Cl-:^Cl]"
        },
        {
          "index": 2,
          "body": "[^Na][^Cl]"
```

```
        }
      ],
      "fragments_defined": [],
      "fragments_used": [],
      "beads": [
        "Cl",
        "Na"
      ],
      "semantic_groups": [],
      "semantic_group_definitions": {}
    },
    "W": {
      "name": "W",
      "resolution": "CG",
      "body": "[ [OOOO:^W] | [^W] ]",
      "layer_count": 2,
      "layers": [
        {
          "index": 1,
          "body": "[OOOO:^W]"
        },
        {
          "index": 2,
          "body": "[^W]"
        }
      ],
      "fragments_defined": [],
      "fragments_used": [],
      "beads": [
        "W"
      ],
      "semantic_groups": [],
      "semantic_group_definitions": {}
    }
  },
  "errors": [],
  "warnings": [],
  "valid": true
}
```

S6. DPPC Bilayer (Atomistic)

```
LiMBS1.0 ||
rsln:AA ||
lsc:1 ||
uls:1 ||
lls:1 ||
{DPPC:tails=16:0-16:0,head=PC,charge=0.0} ||
leaflet:count{-u{DPPC:158}-l{DPPC:158}} ||
type:planar ||
box:[10.0,10.0,8.0]nm ||
sol:TIP3P ||
salt:0.15M NaCl
|++|
DPPC=AA|[
{#T16=CCCCCCCCCCCCCCCC}
[N](C)(C)(C)CCOP(=O)(O)OCCO(=O)[#T16]CO(=O)[#T16]
]
|+|
NaCl=AA|[
[Na+][Cl-]
]
|+|
TIP3P=AA|[
[O]
]
```

This section provides the complete atomistic LiMBS specification for the DPPC bilayer presented in the main manuscript. The #T16 fragment defines the saturated C16 acyl chain used at both lipid-tail positions.

S7. DPPC Bilayer (Coarse-grained)

```
LiMBS1.0 ||
rsln:CG ||
lsc:1 ||
uls:1 ||
lls:1 ||
{DPPC:tails=16:0-16:0,head=PC,charge=0.0} ||
leaflet:count{-u{DPPC:158}-l{DPPC:158}} ||
type:planar ||
box:[10.0,10.0,8.0]nm ||
sol:W ||
salt:0.15M NaCl
|++|
DPPC=CG|[
[N(C)(C)(C)CC:^NC3]
[OP(=O)(O)(O):^PO4]
[CCOC(=O):^GL1]
[COC(=O):^GL2]
[#TailA]
[#TailB]
{#TailA=[>][CCC:^C1A][CCCC:^C2A][CCCC:^C3A][CCCC:^C4A]}
{#TailB=[>][CCC:^C1B][CCCC:^C2B][CCCC:^C3B][CCCC:^C4B]}
|
[^NC3]
[^PO4]
[^GL1]
[^GL2]
[#TailA]
[#TailB]
{#TailA=[>][^C1A][^C2A][^C3A][^C4A]}
{#TailB=[>][^C1B][^C2B][^C3B][^C4B]}
]
|+|
NaCl=CG|[
[Na+:^Na][Cl-:^Cl]
|
[^Na][^Cl]
]
|+|
W=CG|[
[OOOO:^W]
|
[^W]
]
```

This section provides the complete CG LiMBS specification corresponding to the DPPC bilayer described in the main manuscript. Layer 1 records the MARTINI3 atom-to-bead representation, and Layer 2 records the corresponding bead-level topology.

S8. DPPC Bilayer-Super Coarse-Grained (SCG)

```
LiMBS1.0 ||
rsln:SCG ||
lsc:1 ||
uls:1 ||
lls:1 ||
{DPPC:tails=16:0-16:0,head=PC,charge=0.0} ||
leaflet:count{-u{DPPC:158}-l{DPPC:158}} ||
type:planar ||
box:[10.0,10.0,8.0]nm ||
sol:WW ||
salt:0.15M NaCl
|++|
DPPC=SCG|[
[N(C)(C)(C)CC:^NC3][OP(=O)(O)(O):^PO4][CCOC(=O):^GL1][COC(=O):^GL2][#TailA][#
TailB]
{#TailA=[>][CCC:^C1A][CCCC:^C2A][CCCC:^C3A][CCCC:^C4A]}
{#TailB=[>][CCC:^C1B][CCCC:^C2B][CCCC:^C3B][CCCC:^C4B]}
|
[^NC3][^PO4][^GL1][^GL2][#TailA][#TailB]
{#TailA=[>][^C1A][^C2A][^C3A][^C4A]}
{#TailB=[>][^C1B][^C2B][^C3B][^C4B]}
|
[^^HEAD][^^GLYCEROL][^^TAIL]
{^^HEAD=[^NC3][^PO4]}
{^^GLYCEROL=[^GL1][^GL2]}
{^^TAIL=[#TailA][#TailB]}
]
|+|
NaCl=SCG|[
[Na+:^Na][Cl-:^Cl]
|
[^Na][^Cl]
|
[^^CATION][^^ANION]
{^^CATION=[^Na]}
{^^ANION=[^Cl]}
]
|+|
WW=SCG|[
[OOOOOOOO:^WW]
|
[^WW]
|
[^^SOLVENT]
{^^SOLVENT=[^WW]}
]
```

This section presents the three-layer SCG representation for a single-component DPPC membrane. Layer 1 maps atomistic substructures to CG beads. Layer 2 encodes the bead-level topology. Layer 3 uses the ^^ prefix to aggregate beads into named functional domains: {^^HEAD=[^NC3][^PO4]}.

S9. DPPC Vesicle (Coarse-grained)

```
LiMBS1.0 ||
rsln:CG ||
lsc:1 ||
uls:1 ||
lls:1 ||
{DPPC:tails=16:0-16:0,head=PC,charge=0.0} ||
leaflet:ratio{-u{DPPC:1.0}-l{DPPC:1.0}} ||
type:vesicle ||
box:[30.0,30.0,30.0]nm ||
sol:W ||
salt:0.15M NaCl
|++|
DPPC=CG|[
[N(C)(C)(C)CC:^NC3]
[OP(=O)(O)(O):^PO4]
[CCOC(=O):^GL1]
[COC(=O):^GL2]
[#TailA]
[#TailB]
{#TailA=[>][CCC:^C1A][CCCC:^C2A][CCCC:^C3A][CCCC:^C4A]}
{#TailB=[>][CCC:^C1B][CCCC:^C2B][CCCC:^C3B][CCCC:^C4B]}
|
[^NC3]
[^PO4]
[^GL1]
[^GL2]
[#TailA]
[#TailB]
{#TailA=[>][^C1A][^C2A][^C3A][^C4A]}
{#TailB=[>][^C1B][^C2B][^C3B][^C4B]}
]
|+|
NaCl=CG|[
[Na+:^Na][Cl-:^Cl]
|
[^Na][^Cl]
]
|+|
W=CG|[
[OOOO:^W]
|
[^W]
]
```

This section provides the complete CG LiMBS specification for the DPPC vesicle shown in the main manuscript. The leaflet: ratio declaration specifies equal relative compositions for the outer and inner leaflets.

S10. DOPC-Cholesterol (Atomistic)

```
LiMBS1.0 ||
rsln:AA ||
lsc:2 ||
uls:2 ||
lls:2 ||
{CHOL:tails=sterol,head=CHOL,charge=0.0;DOPC:tails=18:1-
18:1,head=PC,charge=0.0} ||
leaflet:count{-u{CHOL:16,DOPC:145}-l{CHOL:16,DOPC:145}} ||
type:planar ||
box:[10.0,10.0,8.0]nm ||
sol:TIP3P ||
salt:0.15M NaCl
|++|
CHOL=AA|[
C[C@H](CCCC(C)C)[C@H]1CC[C@@H]2[C@@]1(CC[C@H]3[C@H]2CC=C4[C@@]3(CC[C@@H](C4)O
)C)C
]
|+|
DOPC=AA|[
{#T18_1=CCCCCCCCC=CCCCCCCCC}
[N](C)(C)(C)CCOP(=O)(O)OCCO(=O)[#T18_1]CO(=O)[#T18_1]
]
|+|
NaCl=AA|[
[Na+][Cl-]
]
|+|
TIP3P=AA|[
[O]
]
```

This section provides the atomistic LiMBS specification for a symmetric planar bilayer composed of DOPC and cholesterol. The chemical block encodes DOPC using fragment notation: `{#T18_1=CCCCCCCCC=CCCCCCCCC}` defines the oleoyl(18:1) acyl chain, referenced at both sn-1 and sn-2 ester linkage positions to encode the di-oleoyl composition. Cholesterol is encoded using stereochemically explicit SMILES notation, with @ and @@ descriptors at all eight chiral centers of the steroid nucleus, explicit ring-closure indices for the fused four-ring system, and the 3β-hydroxyl group (the only polar functional group in cholesterol) encoded as a terminal O.

S11. DOPC-Cholesterol (Coarse-grained)

```
LiMBS1.0 ||
rsln:CG ||
lsc:2 ||
uls:2 ||
lls:2 ||
{CHOL:tails=sterol,head=CHOL,charge=0.0;DOPC:tails=18:1-
18:1,head=PC,charge=0.0} ||
leaflet:count{-u{CHOL:16,DOPC:145}-l{CHOL:16,DOPC:145}} ||
type:planar ||
box:[10.0,10.0,8.0]nm ||
sol:W ||
salt:0.15M NaCl
|++|
CHOL=CG|[
[CCC(O):^ROH] [#TailA] [#TailB]
{#TailA=[>][(C=C)C:^R1][CCC:^R2][CCC:^R3][CC:^R4][CC:^R5][CCC:^R6]}
{#TailB=[>][CCC:^C1][CCCCC:^C2]}
|
[^ROH] [#TailA] [#TailB]
{#TailA=[>][^R1][^R2][^R3][^R4][^R5][^R6]}
{#TailB=[>][^C1][^C2]}
]
|+|
DOPC=CG|[
[N(C)(C)(C)CC:^NC3]
[OP(=O)(O)(O):^PO4]
[CCOC(=O):^GL1]
[COC(=O):^GL2]
[#TailA]
[#TailB]
{#TailA=[>][CCCCC:^C1A][CC(C=C):^D2A][CCCC:^C3A][CCCC:^C4A]}
{#TailB=[>][CCCCC:^C1B][CC(C=C):^D2B][CCCC:^C3B][CCCC:^C4B]}
|
[^NC3]
[^PO4]
[^GL1]
[^GL2]
[#TailA]
[#TailB]
{#TailA=[>][^C1A][^D2A][^C3A][^C4A]}
{#TailB=[>][^C1B][^D2B][^C3B][^C4B]}
]
|+|
NaCl=CG|[
[Na+:^Na][Cl-:^Cl]
|
[^Na][^Cl]
]
|+|
W=CG|[
[OOOO:^W]
```

```
|
[^W]
]
```

For the CG representation, the system block is preserved exactly. Only two fields change: rsln:AA becomes rsln:CG, and sol:TIP3P becomes sol:W. In the CG DOPC chemical block, both oleoyl tails carry D-prefix beads (^D2A, ^D2B) at the second position in each tail fragment, consistent with Martini nomenclature for beads containing a cis double bond. Cholesterol uses the nine-bead Martini3 topology: one hydroxy bead (^ROH), six steroid ring beads (^R1-^R6), and two alkyl tail beads (^C1, ^C2).

S12. Bacterial Outer Membrane of *E. coli* (Atomistic)

```
LiMBS1.0 ||
rsln:AA ||
lsc:6 ||
uls:1 ||
lls:5 ||
{LPS:tails=mixed,head=K12_core_LPS,charge=-8.0;
PVCL2:tails=16:0-18:1D11-16:0-18:1D11,head=CL,charge=-2.0;
PMPE:tails=16:0-16:0,head=PE,charge=0.0;
PMPG:tails=16:0-16:0,head=PG,charge=-1.0;
PVPE:tails=16:0-18:1,head=PE,charge=0.0;
PVPG:tails=16:0-18:1,head=PG,charge=-1.0} ||
leaflet:count{-u{LPS:99}-l{PVCL2:26,PMPE:104,PMPG:13,PVPE:104,PVPG:26}} ||
type:planar ||
box:[13.7,13.7,8.0]nm ||
sol:TIP3P ||
salt:0.15M CaCl2
|++|
LPS=AA|[
{#KDO=OC[C@@H](O)[C@H]1O[C@](O)(C[C@@H](O)[C@H]1O)C(=O)(O)}
{#HEP=OC[C@@H](O)[C@@H]1[C@H](OP(=O)(O)O)[C@@H](O)[C@@H](O)[C@H]O1}
{#GLC=OC[C@H]1O[C@H](O)[C@H](O)[C@@H](O)[C@@H]1O}
{#GAL=OC[C@H]1O[C@H](O)[C@H](O)[C@H](O)[C@@H]1O}
{#LipidA=CCCCCCCCCCCCCC(=O)OC(CCCCCCCCCCC)CC(=O)OC1C(NC(=O)CC(CCCCCCCCCCC)OC(
=O)CCCCCCCCCCC)C(OCC2OC(OP(=O)([O])[O])C(NC(=O)CC(O)CCCCCCCCCCC)C(OC(=O)CC(O)
CCCCCCCCCCC)C2O)OC(CO)C1OP(=O)([O])[O]}
[#LipidA][#KDO][#KDO][#HEP][#GLC][#GLC][#HEP][#HEP][#GLC][#GAL]
]
|+|
PVCL2=AA|[
{#T16=CCCCCCCCCCCCCCCC}
{#T18_1_D11=CCCCCCCCCCC=CCCCCCC}
[#T18_1_D11](=O)OCCO(=O)[#T16]COP(=O)OOCCOCOP(=O)OOCCO(=O)[#T18_1_D11]CO(=O)[
#T16]
]
|+|
PMPE=AA|[
{#T16=CCCCCCCCCCCCCCCC}
[N]CCOP(=O)OOCCO(=O)[#T16]CO(=O)[#T16]
]
|+|
PMPG=AA|[
{#T16=CCCCCCCCCCCCCCCC}
OCCOCOP(=O)OOCCO(=O)[#T16]CO(=O)[#T16]
]
|+|
PVPE=AA|[
{#T16=CCCCCCCCCCCCCCCC}
{#T18_1_D11=CCCCCCCCCCC=CCCCCCC}
[N]CCOP(=O)OOCCO(=O)[#T18_1_D11]CO(=O)[#T16]
]
|+|
PVPG=AA|[
{#T16=CCCCCCCCCCCCCCCC}
```

```
{#T18_1_D11=CCCCCCCCCCC=CCCCCCC}
OCCOCOP(=O)OOCCO(=O)[#T18_1_D11]CO(=O)[#T16]
]
|+|
TIP3P=AA|[
[O]
]
|+|
CaCl2=AA|[
[Ca2+][Cl-][Cl-]
]
```

LPS is encoded using hierarchical fragment notation. {#LipidA} encodes the phosphorylated glucosamine disaccharide backbone with acyl chains. {#KDO} encodes 2-keto-3-deoxyoctonate sugar units. {#HEP} encodes heptose units. {#GLC} and {#GAL} encode glucose and galactose outer core sugars. Fragments are declared before use. Each species block re-declares local fragments independently. $CaCl_2$ is used because Ca^{2+} is biologically required for LPS leaflet integrity in Gram-negative bacterial outer membranes.

S13. Bacterial Outer Membrane of *E. coli* (Coarse-Grained)

```
LiMBS1.0 ||
rsln:CG ||
lsc:4 ||
uls:1 ||
lls:3 ||
{LPS:tails=mixed,head=K12_core_LPS,charge=-8.0;POPE:tails=16:0-
18:1,head=PE,charge=0.0;POPG:tails=16:0-18:1,head=PG,charge=-
1.0;PVCL2:tails=16:0-18:1-16:0-18:1,head=CL,charge=-2.0} ||
leaflet:count{-u{LPS:294}-l{POPE:768,POPG:43,PVCL2:43}} ||
type:planar ||
box:[23.0,23.0,15.0]nm ||
sol:W ||
salt:0.15M CaCl2
|++|
LPS=CG|[
[#LipidA] [#InnerCore] [#OuterCore] [#OAntigen]
{#LipidA=
[OCC:^SN4ar]
[OCCO:^SN6][OP(=O)OO:^D][OCC:^SN4ar]
[OCC:^SN4ar][OCC:^SN4ar][OP(=O)OO:^D]
[N-C(=O)C:^SP3d][OCCO:^SP1r][CO:^SP1]
[CCCC:^C1][CCC:^C1][CCC:^C1][C=O:^SN4a]
[CO:^SP1][CCCC:^C1][CCC:^C1][CCC:^C1]
[N-C(=O)C:^SP3d][COC(=O):^N4a]
[CCCC:^C1][CCCC:^C1][CCC:^C1]
[CCCC:^C1][CCCC:^C1][CCC:^C1]
[C=OC:^SN4a][COC=O:^N4a]
[CCCC:^C1][CCC:^C1][CCC:^C1][CCC:^C1]
[CCCC:^C1][CCCC:^C1][CCC:^C1]
}
{#InnerCore=
[#HEP]
[#KDO]
}
{#HEP=
[CO:^TP1][OCCO:^SP4r][OCCO:^SP1r][CCO:^SN6]
[CO:^TN4r][OCC:^SN4ar][OP(=O)OO:^D]
[OCCO:^SP1r][OCCO:^SP1r][OCC:^SN4ar]
[OP(=O)OO:^D][CO:^TP1][OCCO:^SP1r][OCC:^SN6]
}
{#KDO=
[OCC:^SN4ar][CO:^TP1][OCCO:^SP1r][OCC:^SN4ar]
[O=C(O):^SQ5n][OCC:^SN4ar][O=C(O):^SQ5n]
[OCCO:^SP4r][OCCO:^SP1r][CO:^TP1]
}
{#OuterCore=
[#GLC]
[#GAL]
}
{#GLC=
[CCO:^SN6][OCCO:^SP1r][OCCO:^SP1r]
[CCO:^SN6][OCC:^SN4ar][OCCO:^SP1r]
[CCO:^SN6][OCCO:^SP1r][OCCO:^SP1r]
}
{#GAL=
```

```
[OCC:^SN4ar][OCCO:^SP1r][OCCO:^SP4r]
[OCCO:^SP1r][OCCO:^SP1r][OCCO:^SP4r]
}
{#OAntigen=
[#GALNA]
[#GLCNA]
[#MAN]
[#GLC_repeat]
}
{#GALNA=
[CCO:^SN6][OCCO:^SP1r][OCC:^SN4ar][NC(=O)C:^SP3d]
}
{#GLCNA=
[CCO:^SN6][OCCO:^SP1r][OCC:^SN4ar][NC(=O)C:^SP3d]
}
{#MAN=
[CCO:^SN6][OCCO:^SP1r][OCC:^SN4ar][CCO:^SN6]
[OCCO:^SP1r][OCCO:^SP1r]
}
{#GLC_repeat=
[OCCO:^SP1r][OCCO:^SP4r][OCCO:^SP1r]
}
|
[#OAntigen]
[#OuterCore]
[#InnerCore]
[#LipidA]
]
|+|
POPE=CG|[
[N(C)(C):^NH3]
[OP(=O)(O)(O):^PO4]
[CCOC(=O):^GL1]
[COC(=O):^GL2]
[#TailA]
[#TailB]
{#TailA=[>][CCCCC:^C1A][CC(C=C):^D2A][CCCC:^C3A][CCCC:^C4A]}
{#TailB=[>][CCC:^C1B][CCCC:^C2B][CCCC:^C3B][CCCC:^C4B]}
|
[^NH3]
[^PO4]
[^GL1]
[^GL2]
[#TailA]
[#TailB]
{#TailA=[>][^C1A][^D2A][^C3A][^C4A]}
{#TailB=[>][^C1B][^C2B][^C3B][^C4B]}
]

|+|
POPG=CG|[
[OCCOC:^GL0]
[OP(=O)(O)(O):^PO4]
[CCOC(=O):^GL1]
[COC(=O):^GL2]
[#TailA]
[#TailB]
```

```
{#TailA=[>][CCCCC:^C1A][CC(C=C):^D2A][CCCC:^C3A][CCCC:^C4A]}
{#TailB=[>][CCC:^C1B][CCCC:^C2B][CCCC:^C3B][CCCC:^C4B]}
|
[^GL0]
[^PO4]
[^GL1]
[^GL2]
[#TailA]
[#TailB]
{#TailA=[>][^C1A][^D2A][^C3A][^C4A]}
{#TailB=[>][^C1B][^C2B][^C3B][^C4B]}
]
|+|
PVCL2=CG|[
[OP(O)(O)(O):^PA4] [CCOC:^GL0] [OP(O)(O)(O):^PB4] [CCOC(=O):^GL1]
[COC(=O):^GL2] [CCOC(=O):^GL3] [COC(=O):^GL4] [#TailA] [#TailB] [#TailC]
[#TailD]
{#TailA=[>][CCCC:^C1A][CCCC:^C2A][CC(C=C)C:^D3A][CCCC:^C4A]}
{#TailB=[>][CCC:^C1B][CCCC:^C2B][CCCC:^C3B][CCCC:^C4B]}
{#TailC=[>][CCCC:^C1C][CCCC:^C2C][CCC(C=C):^D3C][CCCC:^C4C]}
{#TailD=[>][CCCC:^C1D][CCCC:^C2D][CCCC:^C3D][CCCC:^C4D]}
|
[^PA4] [^GL0] [^PB4] [^GL1] [^GL2] [^GL3] [^GL4] [#TailA] [#TailB] [#TailC]
[#TailD]
{#TailA=[>][^C1A][^C2A][^D3A][^C4A]}
{#TailB=[>][^C1B][^C2B][^C3B][^C4B]}
{#TailC=[>][^C1C][^C2C][^D3C][^C4C]}
{#TailD=[>][^C1D][^C2D][^C3D][^C4D]}
]
|+|
CaCl2=CG|[
[Ca2+:^Ca][Cl-:^Cl][Cl-:^Cl]
|
[^Ca][^Cl][^Cl]
]
|+|
W=CG|[
[OOOO:^W]
|
[^W]
]
```

This section provides the coarse-grained LiMBS specification of outer membrane of *E. coli.* The LPS is represented in a modular form comprising the O-antigen, outer core, inner core, and lipid A substructures. The O-antigen is defined as a repeating glycan segment composed of galactosamine, glucose, mannose, and galactosamine residues, while the outer core is represented using glucose and galactose fragments. The inner core contains heptose and KDO units, and lipid A is described by the phosphorylated glucosamine disaccharide backbone together with its acyl chains and phosphate substituents. This hierarchical representation preserves the connectivity and composition of the LPS while enabling compact and machine-readable encoding of the full structure.

The #O-Antigen, #Outer-Core, #Inner-Core, and #Lipid-A blocks define reusable substructures that are combined to construct the complete LPS molecule. The connectivity descriptors specify how these fragments are linked, while bead annotations preserve the coarse-grained mapping of each sugar and lipid subunit.

S14. Complex Multicomponent Membrane

To demonstrate the ability of LiMBS to represent chemically diverse membrane systems, we represented a 10-component coarse-grained multicomponent membrane containing sterols and multiple phospholipid classes, including PC, PE, PG, PS, and PA lipids. The system consists of a symmetric planar bilayer with 164 lipids in each leaflet (328 total lipids), including cholesterol and mixed saturated and unsaturated lipid tails. The membrane is solvated using Martini water beads and 0.15 M NaCl.
The complete LiMBS descriptor is provided below, including the system level specification and all species level chemical block definitions for lipid, ion, and solvent components.

```
LiMBS1.0 ||
rsln:CG ||
lsc:10 ||
uls:10 ||
lls:10 ||
{CHOL:tails=sterol,head=CHOL,charge=0.0;DOPE:tails=18:1-
18:1,head=PE,charge=0.0;DOPG:tails=18:1-18:1,head=PG,charge=-
1.0;DOPS:tails=18:1-18:1,head=PS,charge=-1.0;DPPC:tails=16:0-
16:0,head=PC,charge=0.0;POPA:tails=16:0-18:1,head=PA,charge=-
1.0;POPC:tails=16:0-18:1,head=PC,charge=0.0;POPE:tails=16:0-
18:1,head=PE,charge=0.0;POPG:tails=16:0-18:1,head=PG,charge=-
1.0;POPS:tails=16:0-18:1,head=PS,charge=-1.0} ||
leaflet:count{-
u{CHOL:25,DOPE:16,DOPG:15,DOPS:15,DPPC:15,POPA:16,POPC:16,POPE:16,POPG:15,POP
S:15}-
l{CHOL:25,DOPE:16,DOPG:15,DOPS:15,DPPC:15,POPA:16,POPC:16,POPE:16,POPG:15,POP
S:15}} ||
type:planar ||
box:[10.0,10.0,8.0]nm ||
sol:W ||
salt:0.15M NaCl
|++|
CHOL=CG|[
[CCC(O):^ROH] [#TailA] [#TailB]
{#TailA=[>][(C=C)C:^R1][CCC:^R2][CCC:^R3][CC:^R4][CC:^R5][CCC:^R6]}
{#TailB=[>][CCC:^C1][CCCCC:^C2]}
|
[^ROH] [#TailA] [#TailB]
{#TailA=[>][^R1][^R2][^R3][^R4][^R5][^R6]}
{#TailB=[>][^C1][^C2]}
]
|+|
DOPE=CG|[
[NCC:^NH3]
[OP(=O)(O)(O):^PO4]
[CCOC(=O):^GL1]
[COC(=O):^GL2]
[#TailA]
[#TailB]
{#TailA=[>][CCCCC:^C1A][CC(C=C):^D2A][CCCC:^C3A][CCCC:^C4A]}
{#TailB=[>][CCCCC:^C1B][CC(C=C):^D2B][CCCC:^C3B][CCCC:^C4B]}
|
[^NH3]
[^PO4]
[^GL1]
[^GL2]
```

```
[#TailA]
[#TailB]
{#TailA=[>][^C1A][^D2A][^C3A][^C4A]}
{#TailB=[>][^C1B][^D2B][^C3B][^C4B]}
]
|+|
DOPG=CG|[
[OCCOC:^GL0]
[OP(=O)(O)(O):^PO4]
[CCOC(=O):^GL1]
[COC(=O):^GL2]
[#TailA]
[#TailB]
{#TailA=[>][CCCCC:^C1A][CC(C=C):^D2A][CCCC:^C3A][CCCC:^C4A]}
{#TailB=[>][CCCCC:^C1B][CC(C=C):^D2B][CCCC:^C3B][CCCC:^C4B]}
|
[^GL0]
[^PO4]
[^GL1]
[^GL2]
[#TailA]
[#TailB]
{#TailA=[>][^C1A][^D2A][^C3A][^C4A]}
{#TailB=[>][^C1B][^D2B][^C3B][^C4B]}
]
|+|
DOPS=CG|[
[NCC(=O)O:^CNO]
[OP(=O)(O)(O):^PO4]
[CCOC(=O):^GL1]
[COC(=O):^GL2]
[#TailA]
[#TailB]
{#TailA=[>][CCCCC:^C1A][CC(C=C):^D2A][CCCC:^C3A][CCCC:^C4A]}
{#TailB=[>][CCCCC:^C1B][CC(C=C):^D2B][CCCC:^C3B][CCCC:^C4B]}
|
[^CNO]
[^PO4]
[^GL1]
[^GL2]
[#TailA]
[#TailB]
{#TailA=[>][^C1A][^D2A][^C3A][^C4A]}
{#TailB=[>][^C1B][^D2B][^C3B][^C4B]}
]
|+|
DPPC=CG|[
[N(C)(C)(C)CC:^NC3]
[OP(=O)(O)(O):^PO4]
[CCOC(=O):^GL1]
[COC(=O):^GL2]
[#TailA]
[#TailB]
{#TailA=[>][CCC:^C1A][CCCC:^C2A][CCCC:^C3A][CCCC:^C4A]}
{#TailB=[>][CCC:^C1B][CCCC:^C2B][CCCC:^C3B][CCCC:^C4B]}

|
```

```
[^NC3]
[^PO4]
[^GL1]
[^GL2]
[#TailA]
[#TailB]
{#TailA=[>][^C1A][^C2A][^C3A][^C4A]}
{#TailB=[>][^C1B][^C2B][^C3B][^C4B]}
]
|+|
POPA=CG|[
[OP(=O)(O)(O):^PO4]
[CCOC(=O):^GL1]
[COC(=O):^GL2]
[#TailA]
[#TailB]
{#TailA=[>][CCCCC:^C1A][CC(C=C):^D2A][CCCC:^C3A][CCCC:^C4A]}
{#TailB=[>][CCC:^C1B][CCCC:^C2B][CCCC:^C3B][CCCC:^C4B]}
|
[^PO4]
[^GL1]
[^GL2]
[#TailA]
[#TailB]
{#TailA=[>][^C1A][^D2A][^C3A][^C4A]}
{#TailB=[>][^C1B][^C2B][^C3B][^C4B]}
]
|+|
POPC=CG|[
[N(C)(C)(C)CC:^NC3]
[OP(=O)(O)(O):^PO4]
[CCOC(=O):^GL1]
[COC(=O):^GL2]
[#TailA]
[#TailB]
{#TailA=[>][CCCCC:^C1A][CC(C=C):^D2A][CCCC:^C3A][CCCC:^C4A]}
{#TailB=[>][CCC:^C1B][CCCC:^C2B][CCCC:^C3B][CCCC:^C4B]}
|
[^NC3]
[^PO4]
[^GL1]
[^GL2]
[#TailA]
[#TailB]
{#TailA=[>][^C1A][^D2A][^C3A][^C4A]}
{#TailB=[>][^C1B][^C2B][^C3B][^C4B]}
]
|+|
POPE=CG|[
[NCC:^NH3]
[OP(=O)(O)(O):^PO4]
[CCOC(=O):^GL1]
[COC(=O):^GL2]
[#TailA]
[#TailB]
```

```
{#TailA=[>][CCCCC:^C1A][CC(C=C):^D2A][CCCC:^C3A][CCCC:^C4A]}
{#TailB=[>][CCC:^C1B][CCCC:^C2B][CCCC:^C3B][CCCC:^C4B]}
|
[^NH3]
[^PO4]
[^GL1]
[^GL2]
[#TailA]
[#TailB]
{#TailA=[>][^C1A][^D2A][^C3A][^C4A]}
{#TailB=[>][^C1B][^C2B][^C3B][^C4B]}
]
|+|
POPG=CG|[
[OCCOC:^GL0]
[OP(=O)(O)(O):^PO4]
[CCOC(=O):^GL1]
[COC(=O):^GL2]
[#TailA]
[#TailB]
{#TailA=[>][CCCCC:^C1A][CC(C=C):^D2A][CCCC:^C3A][CCCC:^C4A]}
{#TailB=[>][CCC:^C1B][CCCC:^C2B][CCCC:^C3B][CCCC:^C4B]}
|
[^GL0]
[^PO4]
[^GL1]
[^GL2]
[#TailA]
[#TailB]
{#TailA=[>][^C1A][^D2A][^C3A][^C4A]}
{#TailB=[>][^C1B][^C2B][^C3B][^C4B]}
]
|+|
POPS=CG|[
[NCC(=O)O:^CNO]
[OP(=O)(O)(O):^PO4]
[CCOC(=O):^GL1]
[COC(=O):^GL2]
[#TailA]
[#TailB]
{#TailA=[>][CCCCC:^C1A][CC(C=C):^D2A][CCCC:^C3A][CCCC:^C4A]}
{#TailB=[>][CCC:^C1B][CCCC:^C2B][CCCC:^C3B][CCCC:^C4B]}
|
[^CNO]
[^PO4]
[^GL1]
[^GL2]
[#TailA]
[#TailB]
{#TailA=[>][^C1A][^D2A][^C3A][^C4A]}
{#TailB=[>][^C1B][^C2B][^C3B][^C4B]}
]
|+|
NaCl=CG|[
[Na+:^Na][Cl-:^Cl]
|
[^Na][^Cl]
```

```
]
|+|
W=CG|[
[OOOO:^W]
|
[^W]
]
```

S15. Representation of SPICA Membrane System

To demonstrate LiMBS representation using a distinct CG bead convention, we provide the complete LiMBS representation of a SPICA coarse-grained membrane system containing POPC, SSM, and coarse-grained water.

```
LiMBS1.0 ||
rsln:CG ||
lsc:2 ||
uls:2 ||
lls:2 ||
{POPC:tails=16:0-18:1,head=PC,charge=0.0;SSM:tails=18:1-
18:0,head=PC,charge=0.0} ||
leaflet:count{-u{POPC:147,SSM:147}-l{POPC:147,SSM:147}} ||
type:planar ||
box:[10.0,10.0,8.0]nm ||
sol:W ||
|++|
POPC=CG|[
[N(C)(C)(C)CC:^NC]
[OP(=O)(O)(O):^PH]
[CCC:^GL]
[OC(=O):^EST1]
[OC(=O):^EST2]
[#TailA]
[#TailB]
{#TailA=[>][CCC:^C11][CCC:^C12][C(C=C):^C13][CCC:^C14][CCC:^C15][CC:^C16]}
{#TailB=[>][CCC:^C21][CCC:^C22][CCC:^C23][CCC:^C24][CC:^C25]}
|
[^NC]
[^PH]
[^GL]
[^EST1]
[^EST2]
[#TailA]
[#TailB]
{#TailA=[>][^C11][^C12][^C13][^C14][^C15][^C16]}
{#TailB=[>][^C21][^C22][^C23][^C24][^C25]}
]
|+|
SSM=CG|[
[N(C)(C)(C)CC:^NC]
[OP(=O)(O)(O):^PH]
[CC:^GL2]
[CO:^OAD]
[NC(=O):^PEP]
[#TailA]
[#TailB]
{#TailA=[>][(C=C)C:^C11][CCC:^C12][CCC:^C13][CCC:^C14][CCC:^C15]}
{#TailB=[>][CCC:^C21][CCC:^C22][CCC:^C23][CCC:^C24][CCC:^C25][CC:^C26]}
|
[^NC]
[^PH]
[^GL2]
[^OAD]
[^PEP]
```

```
[#TailA]
[#TailB]
{#TailA=[>][^C11][^C12][^C13][^C14][^C15]}
{#TailB=[>][^C21][^C22][^C23][^C24][^C25][^C26]}
]
|+|
W=CG|[
[OOO:^W]
|
[^W]
]
```

S16. Ratio-based Planar Membrane

To demonstrate the leaflet: ratio construction pathway for planar CG membranes, we specified a three component DOPC/POPA/POPE membrane with relative leaflet proportions of 0.5, 0.3, 0.2, respectively, in a 10×10×8 nm^3 box.

```
LiMBS1.0 ||
rsln:CG ||
lsc:3 ||
uls:3 ||
lls:3 ||
{DOPC:tails=18:1-18:1,head=PC,charge=0.0;POPA:tails=16:0-
18:1,head=PA,charge=-1.0;POPE:tails=16:0-18:1,head=PE,charge=0.0} ||
leaflet:ratio{-u{DOPC:0.5,POPA:0.3,POPE:0.2}-l{DOPC:0.5,POPA:0.3,POPE:0.2}}
||
type:planar ||
box:[10.0,10.0,8.0]nm ||
sol:W ||
salt:0.15M NaCl
|++|
DOPC=CG|[
[N(C)(C)(C)CC:^NC3]
[OP(=O)(O)(O):^PO4]
[CCOC(=O):^GL1]
[COC(=O):^GL2]
[#TailA]
[#TailB]
{#TailA=[>][CCCCC:^C1A][CC(C=C):^D2A][CCCC:^C3A][CCCC:^C4A]}
{#TailB=[>][CCCCC:^C1B][CC(C=C):^D2B][CCCC:^C3B][CCCC:^C4B]}
|
[^NC3]
[^PO4]
[^GL1]
[^GL2]
[#TailA]
[#TailB]
{#TailA=[>][^C1A][^D2A][^C3A][^C4A]}
{#TailB=[>][^C1B][^D2B][^C3B][^C4B]}
]
|+|
POPA=CG|[
[OP(=O)(O)(O):^PO4]
[CCOC(=O):^GL1]
[COC(=O):^GL2]
[#TailA]
[#TailB]
{#TailA=[>][CCCCC:^C1A][CC(C=C):^D2A][CCCC:^C3A][CCCC:^C4A]}
{#TailB=[>][CCC:^C1B][CCCC:^C2B][CCCC:^C3B][CCCC:^C4B]}
|
[^PO4]
[^GL1]
[^GL2]
[#TailA]
[#TailB]

{#TailA=[>][^C1A][^D2A][^C3A][^C4A]}
```

```
{#TailB=[>][^C1B][^C2B][^C3B][^C4B]}
]
|+|
POPE=CG|[
[N:^NH3]
[OP(=O)(O)(O):^PO4]
[CCOC(=O):^GL1]
[COC(=O):^GL2]
[#TailA]
[#TailB]
{#TailA=[>][CCCCC:^C1A][CC(C=C):^D2A][CCCC:^C3A][CCCC:^C4A]}
{#TailB=[>][CCC:^C1B][CCCC:^C2B][CCCC:^C3B][CCCC:^C4B]}
|
[^NH3]
[^PO4]
[^GL1]
[^GL2]
[#TailA]
[#TailB]
{#TailA=[>][^C1A][^D2A][^C3A][^C4A]}
{#TailB=[>][^C1B][^C2B][^C3B][^C4B]}
]
|+|
NaCl=CG|[
[Na+:^Na][Cl-:^Cl]
|
[^Na][^Cl]
]
|+|

W=CG|[
[OOOO:^W]
|
[^W]
]
```

S17. Reusable lipid definition and template library

To reduce the amount of manual input required when preparing multicomponent LiMBS specifications, the LiMBS1.0 reference implementation includes a curated registry of reusable lipid definitions together with coarse-grained structural templates. The registry stores lipid identity, lipid class, formal charge, and, where applicable, acyl-chain composition and force-field-specific tail ordering for supported membrane components. These reusable definitions provide a consistent source of lipid metadata and reduce the need to manually re-enter the same lipid information in different membrane systems.

The current registry includes commonly used phosphatidylcholine (PC), phosphatidylethanolamine (PE), phosphatidylserine (PS), phosphatidylinositol (PI), phosphatidylglycerol (PG), phosphatidic acid (PA), diacylglycerol (DG), sphingomyelin (SM), and cholesterol species. Representative and currently registered lipid identifiers are summarized below.

Lipid class	Predefined lipid entries
PA	DOPA, POPA, PIPA, PAPA
PC	POPC, PIPC, PEPC, PAPC, PUPC, DPPC, DOPC, DIPC
PE	POPE, PIPE, PQPE, PAPE, DAPE, PUPE, DUPE, DOPE
PS	POPS, PIPS, PQPS, PAPS, DAPS, DUPS, DOPS
PI	POPI, PIPI, PAPI, PUPI
PG	DOPG, POPG
DG	PODG, PIDG, PADG, PUDG
SM	DPSM, PSM, NSM, OSM, USM
Sterol	CHOL

Each registry entry contains the metadata required to identify the lipid and construct its corresponding LiMBS description. For example, a phosphatidylcholine lipid is represented by its lipid identifier, PC class, two acyl-chain definitions, and formal charge. Lipids with class-specific charge states are stored with the corresponding charge information so that the registry entry remains self-contained.

For coarse-grained systems, the LiMBS implementation also provides reusable structural templates for supported lipid classes. Headgroup templates are defined for PC, PE, PS, PG, PA, PI, DG, and SM, while cholesterol is handled through a sterol-specific CG representation. The template provides the LiMBS atom-to-bead mapping and bead-reference patterns used to assemble the corresponding LiMBS CG chemical block.

The lipid registry is intended to provide reusable definitions for commonly used membrane components rather than to serve as a comprehensive lipid database. Users may add new lipid definitions when a required membrane component is not included in the current registry, provided that the corresponding lipid metadata and resolution-specific structural representation are defined consistently with the LiMBS grammar.
